# Nitro-, Cyano-, and Methylfuroxans, and Their Bis-Derivatives: From Green Primary to Melt-Cast Explosives

**DOI:** 10.3390/molecules25245836

**Published:** 2020-12-10

**Authors:** Alexander A. Larin, Dmitry M. Bystrov, Leonid L. Fershtat, Alexey A. Konnov, Nina N. Makhova, Konstantin A. Monogarov, Dmitry B. Meerov, Igor N. Melnikov, Alla N. Pivkina, Vitaly G. Kiselev, Nikita V. Muravyev

**Affiliations:** 1 Zelinsky Institute of Organic Chemistry, Russian Academy of Sciences, 47 Leninsky Ave., 119991 Moscow, Russia; roby3@mail.ru (A.A.L.); teslatron@yandex.ru (D.M.B.); fershtat@bk.ru (L.L.F.); alkonnoff@yandex.ru (A.A.K.); mnn@server.ioc.ac.ru (N.N.M.); 2 Semenov Federal Research Center for Chemical Physics, Russian Academy of Sciences, 4 Kosygina Str., 119991 Moscow, Russia; k.monogarov@gmail.com (K.A.M.); mmeerov@mail.ru (D.B.M.); igor.n.melnikov@yandex.ru (I.N.M.); alla_pivkina@mail.ru (A.N.P.); kiselev@phys.nsu.ru (V.G.K.); 3 Novosibirsk State University, 1 Pirogova Str., 630090 Novosibirsk, Russia; 4 Institute of Chemical Kinetics and Combustion SB RAS, 3 Institutskaya Str., 630090 Novosibirsk, Russia

**Keywords:** energetic materials, 1,2,5-oxadiazole-2-oxide, furoxan, regioisomerism, melt-cast explosives, thermochemistry, mechanical sensitivity, detonation performance, quantum chemistry

## Abstract

In the present work, we studied in detail the thermochemistry, thermal stability, mechanical sensitivity, and detonation performance for 20 nitro-, cyano-, and methyl derivatives of 1,2,5-oxadiazole-2-oxide (furoxan), along with their bis-derivatives. For all species studied, we also determined the reliable values of the gas-phase formation enthalpies using highly accurate multilevel procedures W2-F12 and/or W1-F12 in conjunction with the atomization energy approach and isodesmic reactions with the domain-based local pair natural orbital (DLPNO) modifications of the coupled-cluster techniques. Apart from this, we proposed reliable benchmark values of the formation enthalpies of furoxan and a number of its (azo)bis-derivatives. Additionally, we reported the previously unknown crystal structure of 3-cyano-4-nitrofuroxan. Among the monocyclic compounds, 3-nitro-4-cyclopropyl and dicyano derivatives of furoxan outperformed trinitrotoluene, a benchmark melt-cast explosive, exhibited decent thermal stability (decomposition temperature >200 °C) and insensitivity to mechanical stimuli while having notable volatility and low melting points. In turn, 4,4′-azobis-dicarbamoyl furoxan is proposed as a substitute of pentaerythritol tetranitrate, a benchmark brisant high explosive. Finally, the application prospects of 3,3′-azobis-dinitro furoxan, one of the most powerful energetic materials synthesized up to date, are limited due to the tremendously high mechanical sensitivity of this compound. Overall, the investigated derivatives of furoxan comprise multipurpose green energetic materials, including primary, secondary, melt-cast, low-sensitive explosives, and an energetic liquid.

## 1. Introduction

The design and synthesis of new organic functional materials are among the most important parts of modern materials science. Among various desired properties, high energy density materials are a long-sought goal for numerous ongoing research programs [1,2,3,4]. Moreover, traditional energetic materials, such as trinitrotoluene (TNT), hexogen (RDX), pentaerythritol tetranitrate (PETN), and nitroglycerine (NG), suffer from inefficient and unsafe methods for their synthesis and limited environmental compatibility [5,6]. Therefore, the search for novel high-energy materials with balanced properties for a wide range of applications, while adhering to strict demands regarding safety and ecology concerns, remains highly urgent.

In general, the current design of energetic materials is driven by two contradictory trends: an increase of energy content and a decrease of hazards associated with the preparation, handling, and storage of energetic compounds [7]. Indeed, combining high density with thermal stability and moderate sensitivity to external stimuli in the same molecular moiety is a tricky issue. To this end, modern synthetic approaches have shifted, e.g., from hydrocarbon backbones to strained N,O-rich heterocycles [8,9]. Among a variety of energetic patterns considered for further functionalization, the 1,2,5-oxadiazole-2-oxide (often referred to as furoxan) ring is indeed prominent due to its attractive oxygen balance, high density of energetic derivatives, and high positive enthalpy of formation [10,11,12,13,14,15,16,17,18]. Although only two positions in the furoxan ring are available for the introduction of energetic substituents, the *N*-oxide regioisomerism is a promising strategy for tuning the detonation performance of furoxan-based energetic materials [19,20,21,22].

**Figure 1:**
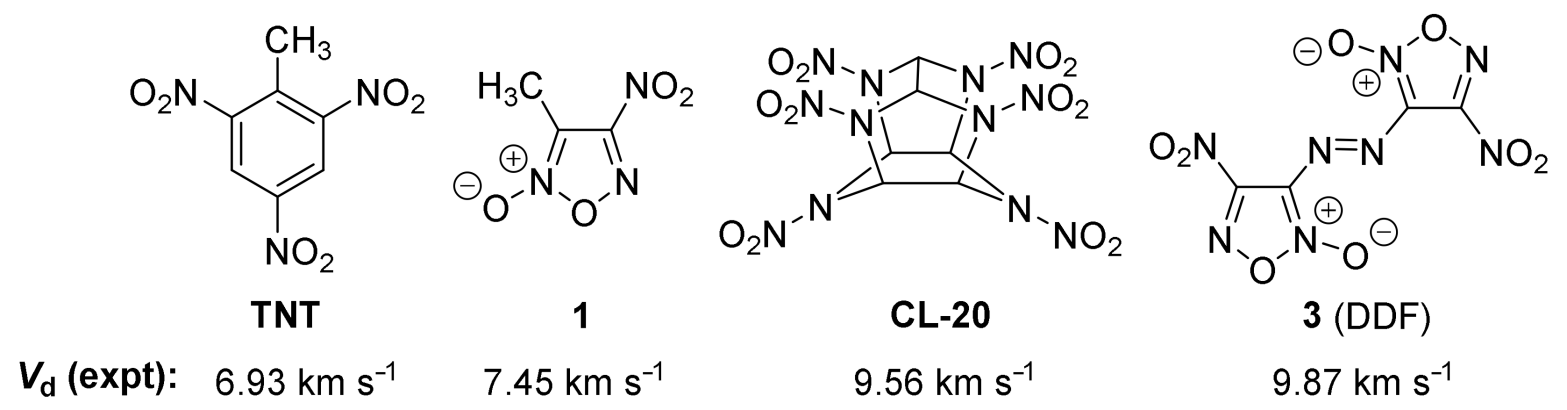
Two promising furoxan-based energetic compounds considered in the present work (**1** and **3**) in comparison with the reference high explosives trinitrotoluene (TNT) and CL-20 along with their experimental velocities of detonation (*V_d_*) [24,25,28,29].

To the best of our knowledge, among a huge variety of energetic furoxan derivatives, the experimentally measured velocities of detonation (*V_d_*) have been reported so far only for two representative species. More specifically, 3-methyl-4-nitrofuroxan (**1**) bears adjacent methyl and nitro groups typical of melt-cast explosives, e.g., a well-known 2,4,6-trinitrotoluene (TNT) or its potential replacement, 2,4-dinitroanisole (DNAN) [23]. Indeed, **1** combines an easily attainable melting point of 67–68 °C and a decent detonation performance evidenced by an experimental *V_d_* of 7.45 km s^−1^ [24]. The latter value exceeds that of the benchmark TNT explosive (6.93 km s^−1^) [25] (Figure 1). Although the melting point of **1** is lower than the temperature range of 80–120 °C typical of melt-cast explosives [23], **1** indeed can be used in low-temperature applications where the high energy content is desirable. Even though the decomposition kinetics of **1** has been studied to some extent [26,27], its phase-change data and mechanical sensitivity have not been reported so far. Apart from this, given the possible tunability of the energetic properties and sensitivity of furoxans provided by *N*-oxide regioisomerism [19,20], the study of the isomeric 3-nitro-4-methylfuroxan (**2**) is also of remarkable interest.

Another energetic furoxan derivative, dinitrodiazenofuroxan (DDF, **3**, Figure 1), is a novel “landmark” explosive with a sound crystal density of 2.002 g cm^−3^ [28]. The reported synthesis of DDF is a laborious multistep procedure with only a 6% overall yield [28]. The experimental *V_d_* of **3** extrapolated to a crystal density at room temperature is extremely high, viz., 9.87 km s^−1^ (Figure 1) [28]. The latter value remains among the highest experimentally measured *V_d_* throughout all organic compounds, e.g., outperforming hexanitrohexaazaisowurtzitane (CL-20), so far the most powerful explosive produced on the industrial scale (Figure 1). 

However, as could be expected, the mechanical sensitivity of **3** is high. In particular, the critical initiation pressure value is 2.2 kbar [30], which is very close to 1.8 kbar of lead azide, a typical primary explosive [31]. The literature data on the thermal stability of **3** are contradictory. Remarkably different DSC decomposition peak temperatures at a ca. 2 K min^−1^ heating rate were reported, i.e., 145 °C [32] and 127 °C [33]. Note that the latter value agrees well with the decomposition without melting at 127–128 °C mentioned earlier in the original work [28]. Therefore, in the present study, we performed a detailed characterization of the thermal stability of DDF.

**Figure 2:**
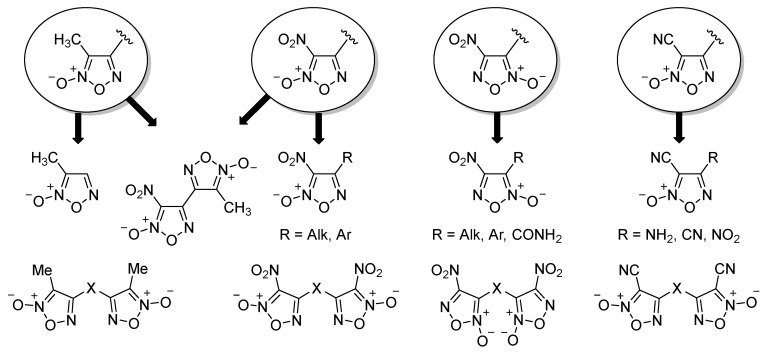
Schematic illustration of the methyl-, nitro-, and cyano-derivatives of furoxan studied in the present work.

A similar idea of a combination of two highly energetic nitrofuroxan moieties has recently been implemented yielding an extremely powerful bisnitrofuroxan [21,34]. In the present study, we consider in detail the important methylfuroxan and nitrofuroxan structural motifs as functional explosophoric building blocks in the design of multipurpose energetic materials (Figure 2). The cyanofuroxan fragment is also scrutinized due to its great importance as a functional moiety in the synthesis of various energetic materials [15,19]. It should be noted that information on the thermal, safety, and energetic properties for nitro-, cyano-, and methylfuroxans remains very limited. At the same time, comprehensive data on a versatile set of these compounds and the in-depth structure–properties relationships are important for the target- and diversity-oriented syntheses of novel advanced energetic materials. Moreover, these data can be useful as a training set for calibration of the neural networks and other machine learning approaches [35,36]. Apart from this, we modified synthetic approaches and reported the missing crystalline structures for some of the species under study.

## 2. Results and Discussion

### 2.1. Synthesis of Furoxan Derivatives

**Scheme 1:**
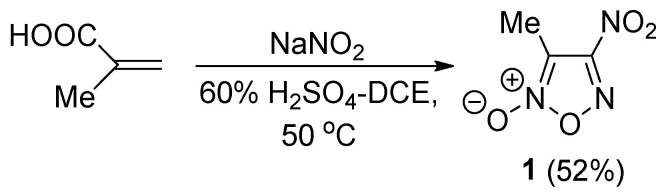
Synthesis of 3-methyl-4-nitrofuroxan **1**.

3-Methyl-4-nitrofuroxan **1** was synthesized through a domino reaction of methacrylic acid with N_2_O_3_ generated in situ from sodium nitrite in acidic media [37]. This approach enables the preparation of the compound **1** in a single synthetic step from cheap and commercially available reagents (Scheme 1).

**Scheme 2:**
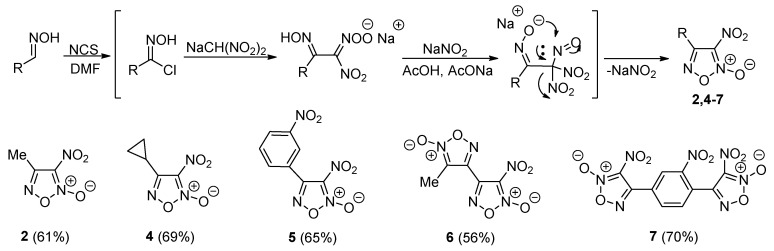
Syntheses of nitrofuroxans **2**, **4**–**7**.

For the synthesis of 3-nitrofuroxans, we used our recently developed approach based on the cascade one-pot reactions of aldoximes. This approach includes the chlorination of initial aldoximes, the acylation of intermediate chloroximes with a dinitromethane sodium salt with a subsequent nitrosation of dinitromethyl derivatives, and in situ intramolecular cyclization [38,39,40]. 3-Nitrofuroxans **2**, **4**–**7** were obtained in high yields in one synthetic step and did not require additional purification (Scheme 2).

**Figure 3:**
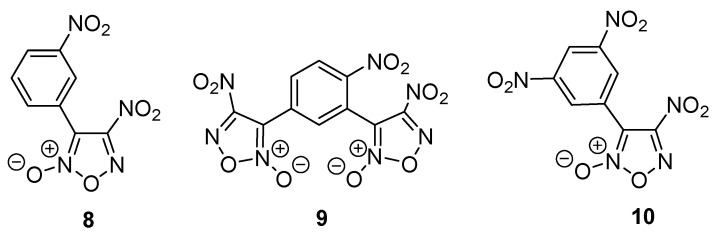
Aryl-substituted nitrofuroxans **8**–**10**.

The isomerization of the corresponding 3-nitrofuroxans afforded 4-nitro isomers **8** and **9** in quantitative yields [39,41]. The compound **8** was additionally nitrated to afford a dinitrophenyl derivative **10** in a yield of 91% (Figure 3).

**Scheme 3:**
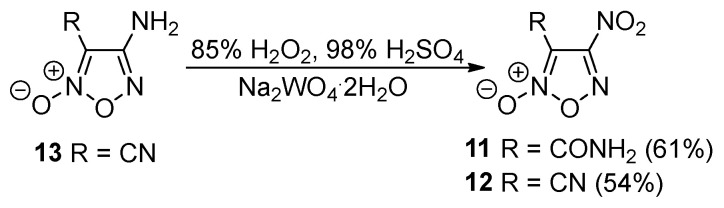
Syntheses of carbamoyl- and cyano-nitrofuroxans **11** and **12**.

The functionally substituted 4-nitrofuroxans **11** and **12** incorporating amide or nitrile moieties were synthesized by an oxidation of the corresponding amines [42,43] (Scheme 3). The oxidation of (carbamoyl)furoxan had been known [43]; however, we improved a synthetic procedure by the addition of Na_2_WO_4_ to an oxidation mixture, which resulted in an increase of the yield of **11**. The synthesis of 3-cyano-4-nitrofuroxan **12** was accomplished previously by the dehydration of 4-nitrofuroxan-3-carboxylic acid amide [44], whereas the oxidation approach provided direct access to the compound **12** through a smaller number of steps starting from the corresponding amine **13**. 

**Scheme 4:**
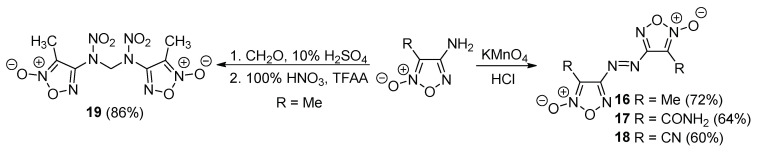
Syntheses of azofuroxans **16**–**18** and a dinitramine-bridged bis-methylfuroxan **19**.

Dicyanofuroxan **14** was synthesized according to a previously published procedure [45]. 3-Methylfuroxan **15** was prepared via nucleophilic substitution of the nitro group in compound **1** by hydride-anion under the action of NaBH_4_ [46]. The oxidative N-N coupling of aminofuroxans afforded a series of symmetrically substituted azo compounds **16**–**18** [43,46]. The tandem Mannich reaction of 4-amino-3-methylfuroxan with subsequent nitration yielded dinitramine **19** [47] (Scheme 4). Dimethylazoxyfuroxan **20** (Figure 2, X: -N(O)=N-) was synthesized through the reductive condensation of **1** [46].

**Figure 4:**
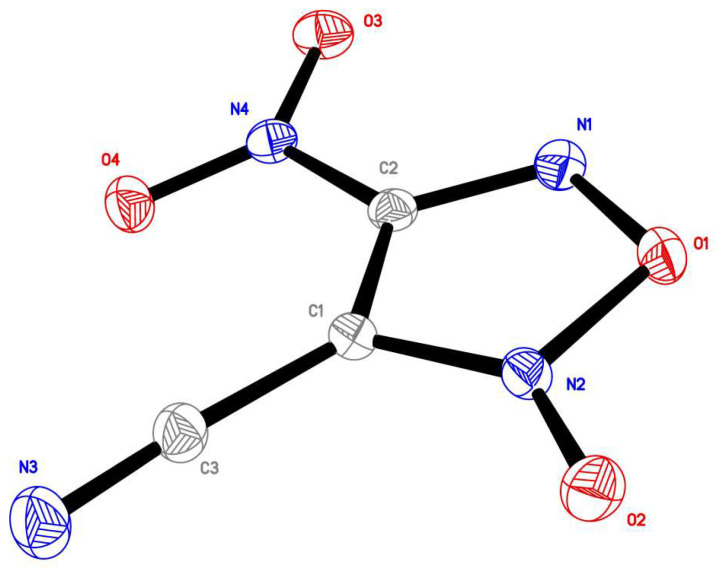
The crystal structure of 3-cyano-4-nitrofuroxan **12**. All atoms are shown as probability ellipsoids of atomic displacements (*p* = 50%).

The purity of all synthesized compounds was determined as 99+% using a set of physicochemical methods including multinuclear (^1^H, ^13^C, ^14^N) NMR spectroscopy, mass spectrometry, and elemental analysis. The structure of 3-cyano-4-nitrofuroxan **12** was additionally confirmed by the X-ray diffraction study (Figure 4). The analysis of structural data revealed the formation of a quite strong intermolecular contact between the O(2) atom of the nitroxyl fragment and the nitro group oxygen atom in **12** (i.e., the O^…^O distance is 2.984 Å), which is responsible for its high density (1.865 g cm^−3^ at 100 K).

### 2.2. Thermal Behavior of the Furoxan Derivatives: Melting and Decomposition

Upon linear heating, most of the analyzed compounds exhibit the three features registered by differential scanning calorimetry (DSC)—viz., melting, thermal isomerization, and thermal decomposition. These three processes will be discussed separately for all compounds studied. 

**Figure 5:**
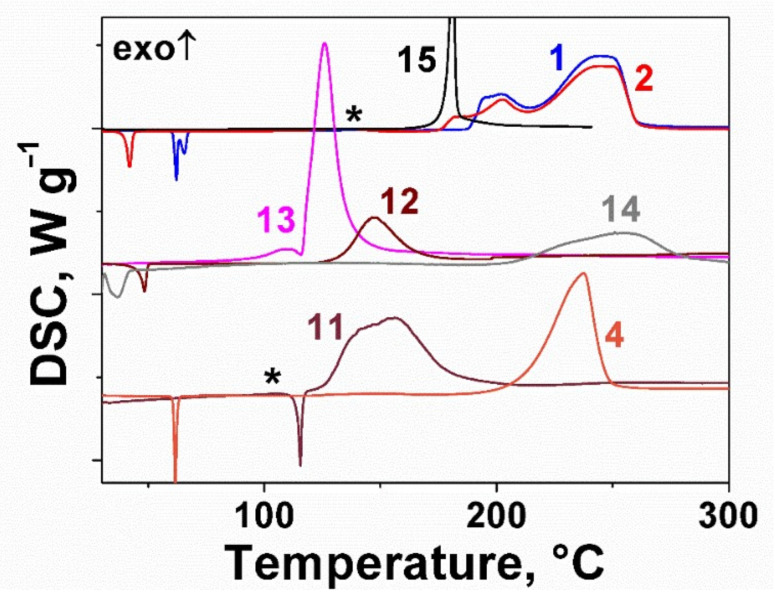
Thermal stability of monocyclic furoxans **1**, **2**, **4**, **11**–**15**. The differential scanning calorimetry (DSC) curves were recorded at a heating rate of 5 K min^−1^. The stars indicate a weak heat release due to the thermal isomerization of **2** and **4**.

Melting was observed for all monocyclic and aryl-substituted furoxans, except **15**, which is liquid at room temperature. The respective melting points are given in Section 2.5, and the raw DSC data for all compounds are shown in Supplementary Materials (Section S2). In general, the melting of monocyclic species **1**, **2**, **4**, **5**, **12**, and **14** occurs in the temperature range of 39–65 °C (Figure 5). The introduction of the amino-group increases the melting temperature to 114 and 117 °C for **11** and **13**, respectively. It is worth noting that 3-methyl-4-nitrofuroxan (**1**) exhibits two resolved endothermic peaks (Figure 5), although the compound has no impurities detectable by the conventional physicochemical methods employed here. To explain this behavior, the sample was heated up to melting, cooled, and reheated (Figure S1, Supplementary Materials). During the second heating cycle, only a single endothermic peak was observed at 65 °C. This value is believed to be a true melting temperature for **1**.

**Figure 6:**
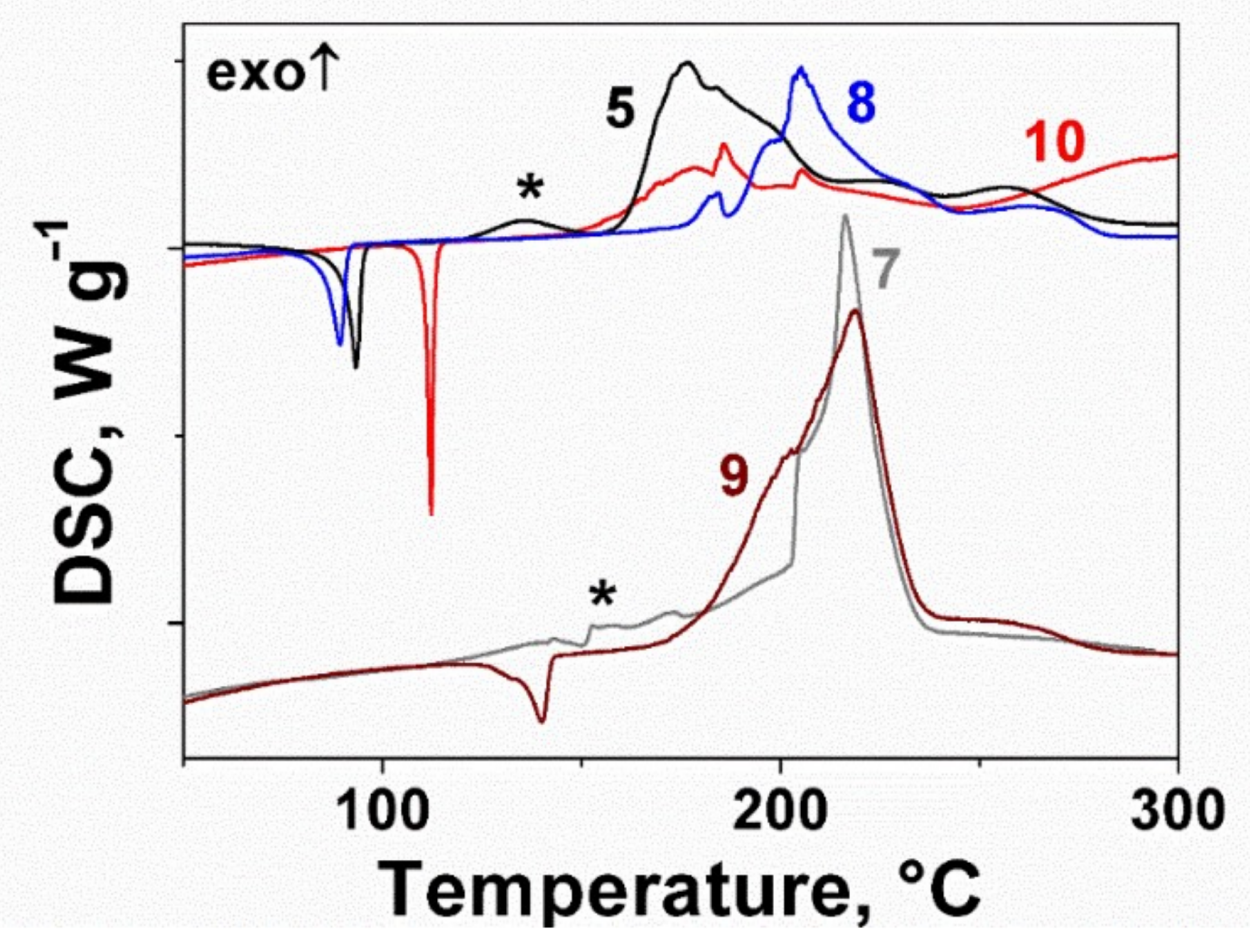
Thermal stability of aryl-furoxans **5** and **7**–**10**. The differential scanning calorimetry (DSC) curves were recorded at a heating rate of 5 K min^−1^. The star indicates a weak heat release due to the thermal isomerization of **5** and **7**.

The aryl-substituted furoxans **5**, **8**, and **10** melt at higher temperatures, i.e., in the range of 83–111 °C, compared to monocyclic nitrofuroxans **1**, **2, 4**, **5**, **12**, and **14** (Figure 6). Note that the nitrophenyl-bridged 4-nitro furoxan **9** undergoes melting, whereas the isomeric **7** does not. Among the bridged bicyclic furoxans, only 3-methyl-3’-nitro-4,4′-bifuroxan **6** and dimethylazoxyfuroxan **20** exhibit visible melting endotherms. The melting point of the latter compound is 173 °C, which is the highest value within the set of the furoxans considered.

**Figure 7:**
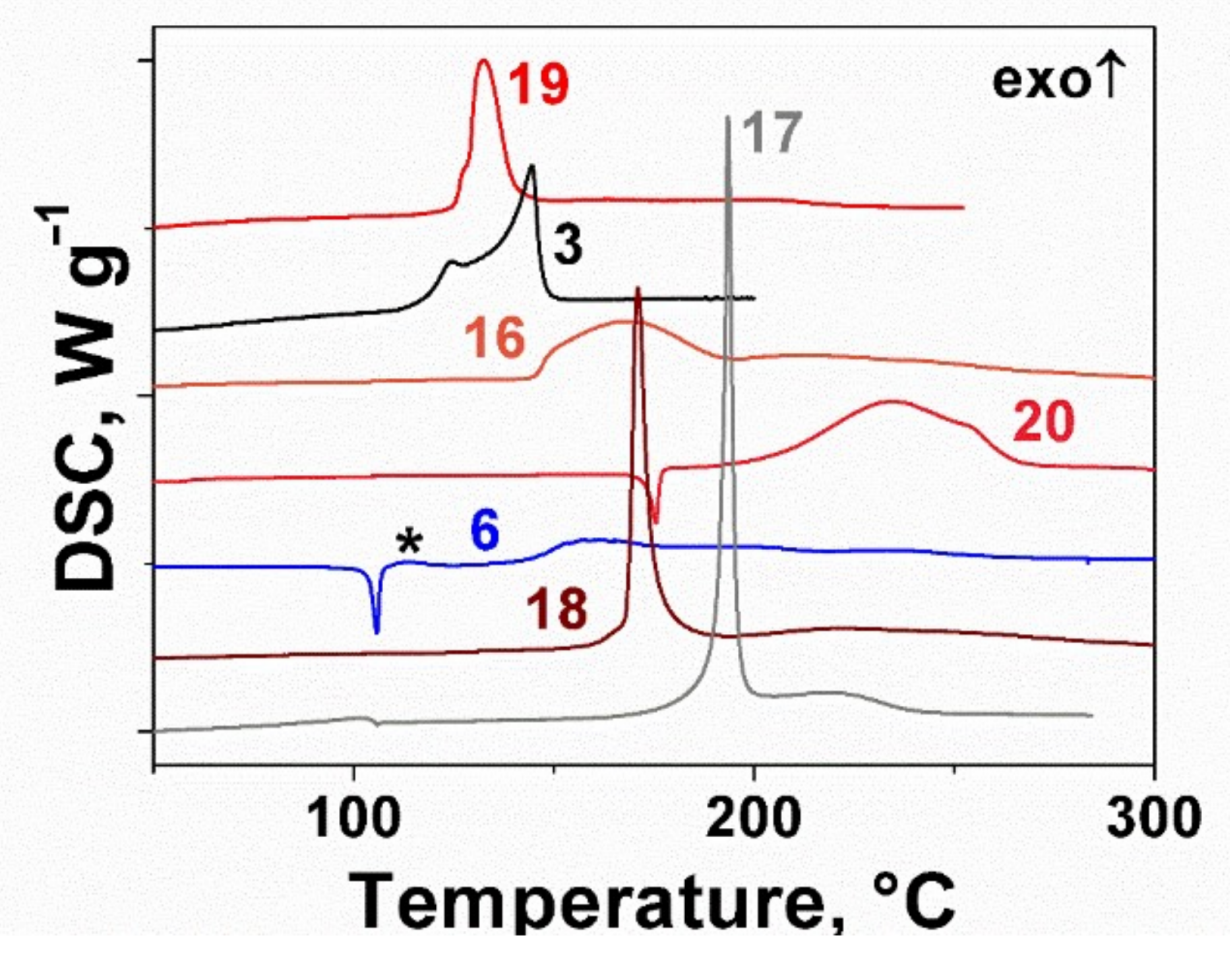
Thermal stability of the bicyclic furoxans **3**, **6**, and **16**–**20**. The differential scanning calorimetry (DSC) curves were recorded at a heating rate of 5 K min^−1^. The star indicates a weak heat release due to a thermal isomerization of **6**.

It is interesting to note that all monocyclic 3-nitrofuroxans **2**, **4**, and **5**–**7** exhibit weak exothermic features on the DSC curves starting at 110–120 °C (indicated by stars in Figure 5, Figure 6 and Figure 7). A detailed analysis of this process is complicated either due to the vaporization of the respective compounds (in the case of **2** and **4**) or the overlap with the thermal decomposition peaks (**5**–**7**). We hypothesized these peaks to represent the thermal isomerization of 3-nitrofuroxans to the thermodynamically more preferable 4-nitro isomers. This reaction is known to occur at elevated temperatures (*t* > 100 °C) for a wide set of nitrofuroxans [10,19,20,21]. 

The third thermal process tracked by DSC is the exothermal thermal decomposition. For several compounds under study, the vaporization rate is high and the broad endotherm of evaporation is observed instead of the exothermic peak of thermal decomposition. This behavior is common for energetic materials and previously some of us proposed the pressure DSC method as a convenient tool to suppress the evaporation and observe the sole thermal decomposition process [48,49]. The elevated pressure DSC curves for the isomeric methylnitrofuroxans **1** and **2** are shown inFigure S2 (Supplementary Materials). Figure 2, Figure 3 and Figure 4 show a comparison of the decomposition exotherms for the investigated compounds, i.e., in some cases the pressure is atmospheric, otherwise the elevated pressure was used to suppress the vaporization (details are given in Section S2 of the Supplementary Materials).

The methyl-substituted furoxans **1**, **2**, and **15** exhibit a similar thermal stability that can be estimated using the extrapolated decomposition onset temperature of 180 °C. The cyanofuroxans **12**–**14** demonstrate much more scattered results—from a thermally stable dicyanofuroxan **14** with the decomposition onset of 204 °C to 4-amino-3-cyanofuroxan **13** with that as low as 96 °C (Figure 5). 3-Cyano-4-nitrofuroxan **13** and 3-carbamoyl-4-furoxan **11** exhibit slightly better thermal stability than that for **13**. The introduction of a cyclopropyl substituent improves the thermal stability of **4** to a level above 200 °C, which is acceptable for applications (Figure 5).

Three nitrophenyl furoxans **5**, **8**, and **10** show a complex thermal decomposition profile with onset temperatures within the range of 150–175 °C. The two nitrofuroxans linked by a nitrophenyl bridge **7** and **9** decompose at temperatures higher than 170 °C with a complex shape of the corresponding heat flow traces (Figure 6). 

Among the bicyclic structures, 3-methyl-3’-nitro-4,4′-bifuroxan **6** has the lowest thermal stability, which is evidenced by an exothermic peak after the melting at 103 °C (Figure 7). The comparison of **16** and **20** shows that the azoxy bridge significantly improves the thermal stability of **20** compared to its counterpart with the azo bridge **16**. The symmetric bicyclic species with carbamoyl (**17**) and cyano (**18**) functionalities exhibit sharp DSC peaks along with those acceptable for application decomposition onsets above 150 °C (Figure 7). Finally, DDF (**3**) releases a large amount of heat upon thermolysis. However, the use of samples as low as 0.16 mg allowed us to obtain a smooth DSC curve with a clear two-stage decomposition behavior (Figure 7). The overall decomposition process starts above 117 °C, and the maximal rate of the heat release is registered at 145 °C. Thus, the literature discrepancy of the decomposition peak temperatures of **3** is resolved: the dominant second stage was observed in [32], whereas the authors of [33] track only the first stage that developed into thermal explosion due to the high sample mass used. 

### 2.3. Thermal Behavior of the Furoxan Derivatives: Vaporization 

**Figure 8:**
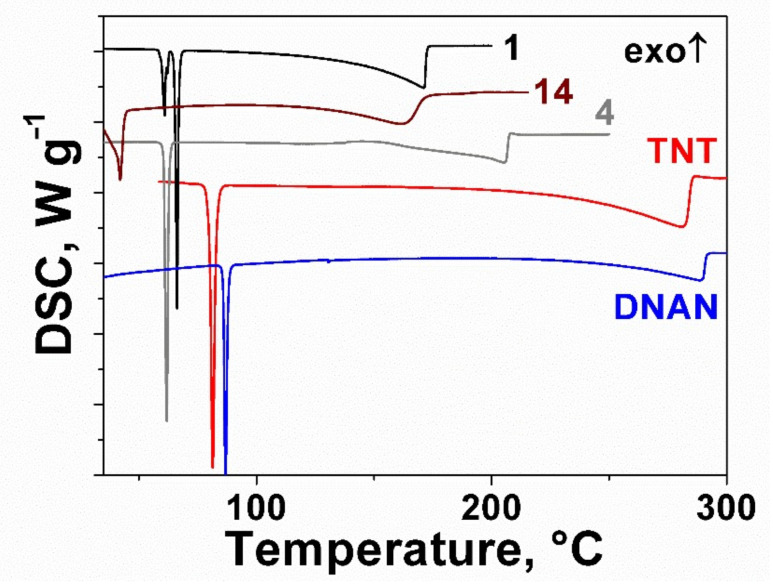
DSC profiles illustrating the melting and subsequent evaporation of selected furoxans **1**, **4**, **14** and the reference energetic materials TNT and 2,4-dinitroanisole (DNAN). The heating rate is 5 K min^−1^.

As mentioned in the Introduction (Section 1), some furoxan derivatives (namely **1**, **4**, and **14**) are considered to be possible replacements to trinitrotoluene in the melt-cast compositions. Therefore, we compared their thermal behavior at atmospheric pressure with the reference melt-cast compounds TNT and DNAN (Figure 8). The prospects of **1** and **14** [23] as melt-cast explosives were considered before, and 3-nitro-4-cyclopropylfuroxan **4** also has a beneficially broad separation of melting and decomposition processes (61 °C and 211 °C, respectively, Figure 8). Note that the temperature of the DSC endothermic evaporation peak can be used for semiquantitative estimations. This temperature is generally close to the boiling point of a species [49] (for a more rigorous determination of the boiling point with DSC, a specific restriction has to be applied to the pan lid orifice along with modified experimental conditions) [50]. As seen from Figure 8, the compounds studied are arranged in the following row of this characteristic temperature: **14** (161 °C) ≈ **1** (171 °C) < **4** (206 °C) < TNT (281 °C vs. the estimated boiling point of 300 ± 10 °C [51]) ≈ DNAN (289 °C). 

Therefore, at the time being, we infer that the high volatility is the main problem hindering the application of **1**, **4**, and **14** as standalone melt-cast explosives. This feature is attributed to the absence of strong intra- and intermolecular interactions (e.g., hydrogen bonds or π-stacking) in the structural motif of the species studied. The previous structural investigations demonstrated that most of the furoxan derivatives have only short O⋯O contacts that do not provide sufficient stabilization via a supramolecular organization [20,52]. The possible ways to resolve this issue could be: (i) the design of novel materials with high-molar mass substituents capable of formation of strong intermolecular bonds to decrease the volatility (cf. **1** and **4**); (ii) the use of lower amounts of **1**, **4**, and **14** in the formulations with a proper selection of other components; (iii) the strict temperature control of the manufacturing process. Apart from this, the melting points of **1**, **4**, and **14** (Figure 8) are notably lower than the temperature range of 80–120 °C typical of melt-cast explosives [23]. However, these compounds still can find their room in low-temperature applications and proper compositions.

### 2.4. Gas-Phase Formation Enthalpies of Furoxan, Its Monocyclic, and Bis-Derivatives 

The solid-state formation enthalpy and density of a particular energetic material are the key input parameters for computational models of detonation performance. Due to natural hindrances with the combustion calorimetry of high-energy compounds, solid-state formation enthalpies are often calculated using quantum chemical values for the gas-phase thermochemistry and empirical estimations for sublimation enthalpy. The latter values generally lie in the narrow range on the enthalpic scale, while the former values are prone to error accumulation and require highly accurate levels of theory [53].

**Figure 9:**
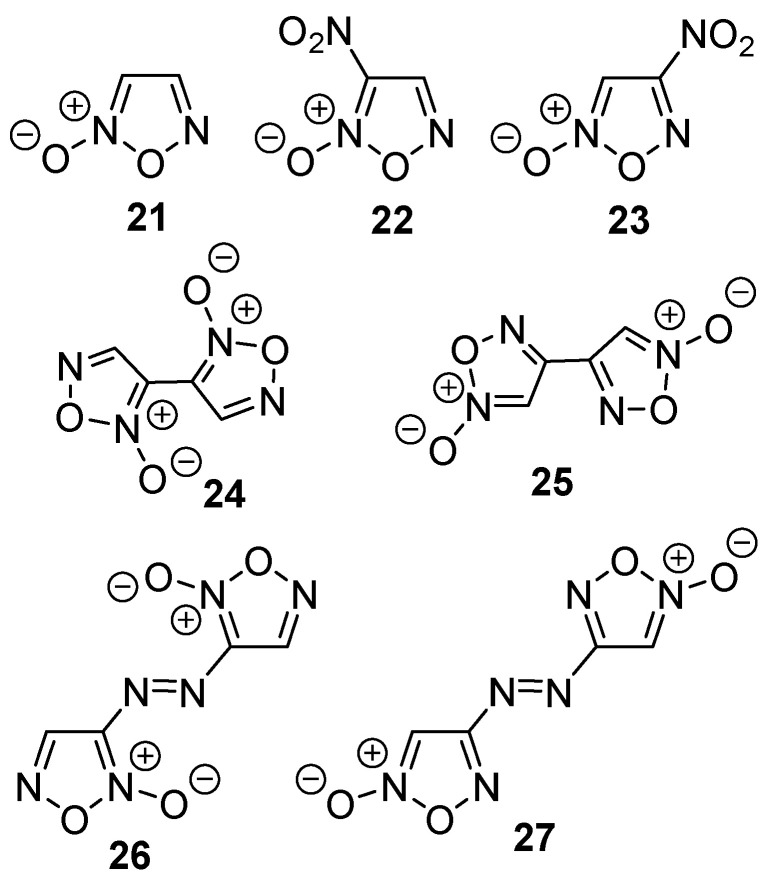
The parent furoxan species (**21**), its nitro derivatives (**22**, **23**), and bis- (**24**, **25**) and azobis bicyclic derivatives (**26**, **27**) considered in the thermochemical calculations.

To this end, earlier, some of us proposed a “bottom-up” approach, that is, the multilevel procedures W2-F12 and/or W1-F12 in conjunction with the atomization energy approach for smaller species complemented with the domain-based local pair natural orbital coupled cluster (DLPNO-CCSD(T)) enthalpies of isodesmic reactions for medium-sized compounds [54,55]. The overall accuracy of these methods on a test set composed of nitrogen-rich heterocyclic compounds turned out to be close to “chemical” (~4 kJ mol^−1^). Thus, we commenced the study of the gas-phase thermochemistry of the furoxan derivatives **1**–**20** from the multilevel calculations of atomization energies for furoxan (**21**), its hypothetical mononitro derivatives **22** and **23,** and several related heterocyclic frameworks (3,3′- and 4,4′-bisfuroxans **24** and **25**, as well as 3,3′- and 4,4′-azobisfuroxans **26** and **27**, Figure 9).

**Scheme 5:**
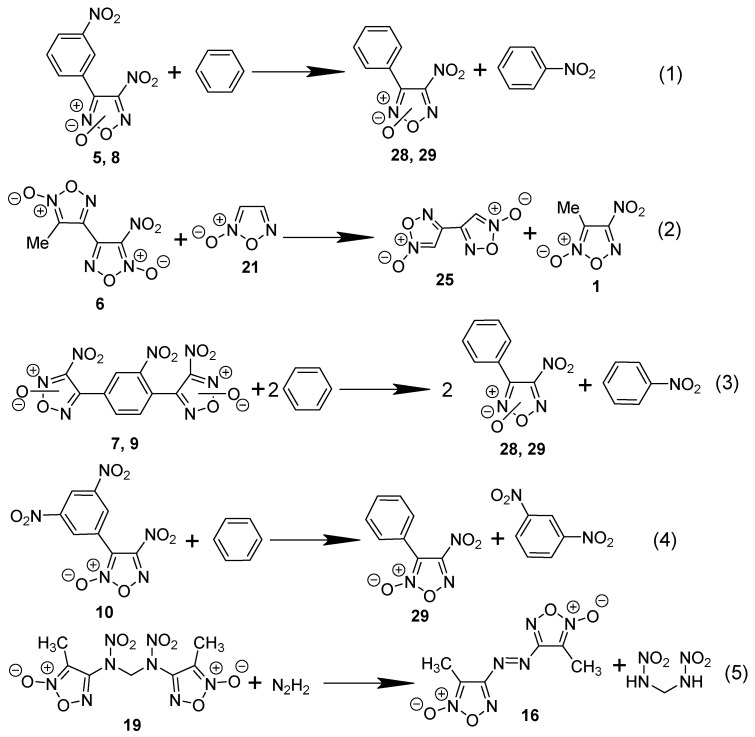
The isodesmic reactions employed in the present work to calculate the formation enthalpies Δ*_f_H*^0^(*g*) of the species **5**–**10** and **19**.

The most reliable W2-F12 formation enthalpies for **21**–**27** are listed in Table 1. By exploiting molecular point group symmetries, we drastically reduce the computational time and resources needed to perform heavy multilevel calculations. For several species (e.g., **3**, **17**, **18**, **20**), we combined the W1-F12 atomization energies for a symmetrical less favorable energetically conformer with the DLPNO-CCSD(T) enthalpy difference between conformers. Note that the (carbamoyl)nitro (**11**) and dicyano derivatives of monocyclic furoxan (**14**) are the largest species, for which the W2-F12 calculations are feasible, and the azobis derivatives of furoxan **3** and **17** are at the limit of the W1-F12 feasibility, respectively. Thus, in order to consider the nitrobenzene derivatives **5** and **8**–**10**, the methylnitro compound **6**, and dinitramine-bridged bis-methylfuroxan **19**, we used a “bottom-up” ab initio computational approach. Namely, we proposed a series of isodesmic reactions of the target compounds **6**, **8**, and **19** with simpler species (Scheme 5). Moreover, to reduce another source of computational uncertainties, the reactions (1)–(4) (Scheme 5) were designed to be homodesmotic (i.e., those conserving the number of carbon and nitrogen atoms in corresponding states of hybridization) [56]. This minimizes the error due to differences in the ring strain and cyclic delocalization. The enthalpies of these reactions are calculated with the DLPNO-CCSD(T) technique, and the formation enthalpies of all simpler species are calculated using the atomization energy approach at the W1-F12 level of theory. The most reliable calculated gas-phase formation enthalpies for the species **1**–**20** along with computational details are summarized in Table 1.

Note that the W2-F12 gas-phase formation enthalpies for the monocyclic furoxan and bicyclic furoxan derivatives (**21**–**23**, **1**, **2**, **11**–**15**, Table 1) can be used for reliable thermochemical estimations using the “bottom-up” approach [55] for a huge variety of novel furoxan derivatives.

### 2.5. Detonation Performance 

Having obtained the gas-phase formation enthalpies for the species **1**–**20**, we complemented them with the sublimation enthalpies $\Delta_{sub}H^{0}$ estimated with the use of the Westwell formula (often referred to as Trouton’s rule) [57]. The resulting solid-state formation enthalpies $\Delta_{f}H^{0}\left(s\right)$ are shown in Table 2. To estimate the accuracy of our computational approach, it is instructive to compare the calculated $\Delta_{f}H^{0}\left(s\right)$ values with the available experimental data. However, the reliable experimental value is reported only for **1**: $\Delta_{f}H^{0}\left(s\right)$ = 100.8 ± 0.8 kJ mol^−1^ [24]. For three other species from the present study, viz., **3**, **12**, and **14**, the ICT database [58] suggests the following values of $\Delta_{f}H^{0}\left(s\right)$: 667.8, 380.7, and 465.3 kJ mol^−1^, respectively. However, it is not clear whether these data are true experimental values or they were estimated using the group increment method. The comparison of these data with those calculated in the present work (123, 725, 361, and 495 kJ mol^−1^ for **1**, **3**, **12**, and **14**, respectively) shows that the discrepancy is less than 25 kJ mol^−1^ for all species except **3**. The main source of uncertainties in the $\Delta_{f}H^{0}\left(s\right)$ values is obviously the use of empirical estimations of the sublimation enthalpies $\Delta_{sub}H^{0}$. We also compared the empirical values of $\Delta_{sub}H^{0}$ with the experimental values for **1**, **2**, and **13** obtained from thermogravimetry experiments [59]. In line with the above-discussed uncertainties, the average discrepancy was around 20 kJ mol^−1^. 

It should be emphasized that we aimed foremost at the reliable estimations of the detonation performance of the species studied. The use of the two enthalpies of formation values for **1**, **3**, **12**, and **14**, calculated in the present study and those from literature, leads to the errors less than 0.15 km s^−1^ for detonation velocities and less than 0.2 kJ g^−1^ for heats of explosion. Furthermore, the available experimental values of the detonation velocities (*V_D_*) for **1** and **3** also justify this assumption. More specifically, the experimental *V_D_* of **1** is 7.45 km s^−1^ (the sample density 1.60 g cm^−3^) [24], which agrees well with the calculated value of 7.56 km s^−1^. In the case of DDF (**3**), the calculated detonation velocity of 10.0 km s^−1^ is also close to the reported experimental values 9.78 km s^−1^ [33] and 9.87 km s^−1^ [28]. Therefore, the errors due to the uncertainties in the $\Delta_{f}H^{0}\left(s\right)$ and the computational methodology employed are quite low and allow for reliable evaluation of the energetic performance of the furoxan derivatives studied. Thus, the computed detonation velocities of **1**–**20** are shown in Table 2.

The isomeric methylnitrofuroxans **1** and **2** exhibit close detonation parameters, even though **2** has a slightly higher density and formation enthalpy. These two compounds as well as other monocyclic nitro (**4**, **11**, **12**) and cyano-derivatives of furoxan (**13**, **14**) outperform the benchmark trinitrotoluene both in terms of the detonation velocity and heat of explosion (Table 2). 3-Methylfuroxan **15** is a surprisingly stable liquid with a moderate detonation velocity of 6.1 km s^−1^. Its energetic properties are inferior to those for nitroglycerin, but thermal stability and sensitivity are much better (Table 1). In the case of bicyclic furoxan derivatives **16**–**20**, it is reasonable to compare their detonation performance with that of the commonly used secondary explosive pentaerythritol tetranitrate (PETN, Table 2). The two azo-bridged furoxans **17** and **18** exhibit the close values of *V_D_* with slightly better sensitivity without melting, which is typical of PETN. The extremely high explosive performance of **3** is remarkable; however, this comes at the cost of high sensitivity (vide infra). The impact and friction sensitivity values of **3** are close to those of common primary explosives (e.g., lead azide).

### 2.6. Sensitivity to Mechanical Stimuli 

**Figure 10:**
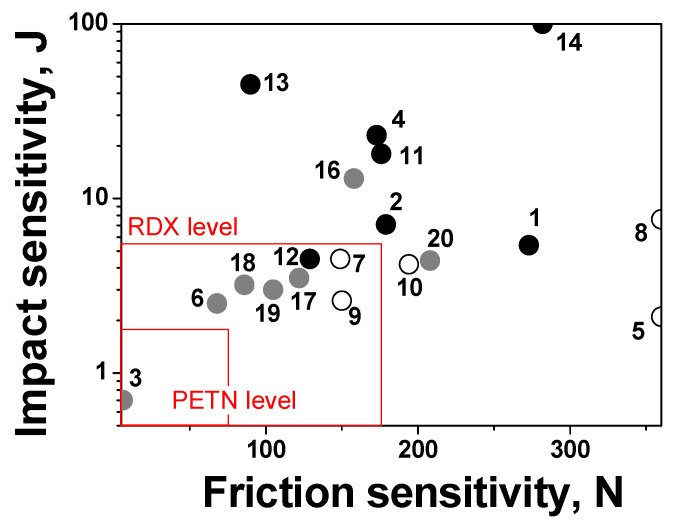
The impact and friction sensitivities of the species under study (**1**–**20**). The whole dataset of the mechanical sensitivity values, including the uncertainties, is given in Table S6 (Supplementary Materials). The filled black circles correspond to the monocyclic species, the empty circles—to the benzene derivatives of furoxan, and the gray-filled circles—to the bicyclic derivatives.

The impact (IS) and friction sensitivity (FS) values for the investigated compounds are summarized in Table 2 and Figure 10. Most of the species studied exhibit high impact sensitivity, viz., below the level of nitramines (8 J). The clear outlier is dicyanofuroxan **14,** which remains intact at the maximal drop energy of 100 J. Note, however, that **14** has a very low melting point of 39 °C. The errors associated with the low-melting-point samples have been mentioned in a recent review of the sensitivity test methodology [63]. The aminocyanofuroxan **13** also exhibits a low impact sensitivity, but its FS value is very low (Table 2, Figure 10). Another group of the compounds clustered in the region IS ~15 J includes **4**, **11**, and **16**. This level corresponds to explosives with moderate sensitivity (e.g., 14 J in the case of tetryl). The friction sensitivity exhibits a higher span (Figure 10) from an extremely sensitive **3** (FS = 6 N) to insensitive **5** and **8** (less than 50% of explosions at the maximal accessible load of 360 N).

## 3. Experimental and Computational Methods

All reactions were carried out in well-cleaned oven-dried glassware with a magnetic stirring. ^1^H and ^13^C NMR spectra were recorded on a Bruker AM-300 (300.13 and 75.47 MHz, respectively) spectrometer and referenced to a residual solvent peak. ^14^N NMR spectra were measured on a Bruker AM-300 (21.69 MHz) spectrometer using MeNO_2_ (δ_14N_ = 0.0 ppm) as an external standard. The chemical shifts are reported in ppm (δ); the multiplicities are indicated by s (singlet), d (doublet), t (triplet), q (quartet), m (multiplet), and br (broad). The coupling constants *J* are reported in Hertz. Elemental analyses were performed by the CHN Analyzer Perkin-Elmer 2400. The high-resolution mass spectra were recorded on a Bruker microTOF spectrometer with electrospray ionization (ESI). All measurements were performed in a positive (+MS) ion mode (interface capillary voltage: 4500 V) with scan range *m/z*: 50–3000. External calibration of the mass spectrometer was performed with Electrospray Calibrant Solution (Fluka). A direct syringe injection was used for all analyzed solutions in MeCN (a flow rate: 3 μL min^−1^). Nitrogen was used as a nebulizer gas (0.4 bar) and as a dry gas (4.0 L min^−1^); the interface temperature was set at 180 °C. All spectra were processed with the Bruker DataAnalysis 4.0 software package. Analytical thin-layer chromatography (TLC) was carried out on Merck 25 TLC silica gel 60 F_254_ aluminum sheets. The visualization of the TLC plates was accomplished with UV light. The densities were measured with a Micromeritics AccuPyc II 1340 gas pycnometer.

The thermal stability of the species studied was monitored using DSC 204 HP apparatus (Netzsch). The samples of 0.5–1.5 mg weight were linearly heated up to a 350 °C at 5 K min^−1^ rate under a nitrogen flow. Most of the experiments were run under atmospheric pressure, but for several highly volatile compounds, the elevated pressure of 2.0 MPa was applied. This methodology (referred to as the pressure DSC) is discussed in detail elsewhere [48,49].

Electronic structure calculations were performed using the Gaussian 09 [64], Molpro 2015 [65], and ORCA 4.0 [66] program packages. The gas-phase enthalpies of formation (at *p*^0^ = 1 bar and *T* = 298.15 K, Δ*H*_f_^0^(g)) were calculated using the explicitly correlated W1-F12 and W2-F12 multilevel procedures [67] and the atomization energy and isodesmic reaction approaches described in detail elsewhere [55,68]. Note that the W1-F12 procedure employed in the present work had been slightly modified in comparison with the originally proposed technique: namely the B3LYP-D3BJ/def2-TZVPP optimized geometries (the ZPE correction factor of 0.99) were used [69,70] and the diagonal Born–Oppenheimer corrections were omitted. The multireference character of the wave functions of the reagents, intermediates, and transition states considered in the present work was estimated using the T1 diagnostic for the CCSD calculation [71]. The modest T1 values obtained in all cases (<0.025) justify the reliability of the single reference-based electron correlation procedure employed in the present study. Apart from this, to estimate the possible contributions from post-CCSD(T) excitations to the valence component of the atomization energies, the %TAE[(T)] diagnostics (viz, the percentage of the perturbative triples (T) in the CCSD(T) atomization energy) was employed [72]. For all species studied, the latter value did not exceed 5%. This fact also justifies the reliability of the CCSD(T) atomization energies calculated in the framework of the W1-F12 procedure. The heats of formation at 0 K for the elements in the gas phase Δ_f_*H*_gas_^0K^(C) = 169.98 kcal mol^−1^, Δ_f_*H*_gas_^0K^(H) = 51.63 kcal mol^−1^, Δ_f_*H*_gas_^0K^(N) = 112.53 kcal mol^−1^, and Δ_f_*H*_gas_^0K^(O) = 58.99 kcal mol^−1^ were taken from the NIST-JANAF tables [73].

For several isodesmic reactions, single-point electronic energies were calculated using the DLPNO-CCSD(T) methodology (the “NormalPNO” truncation thresholds were set) [74] along with the aug-cc-pVQZ basis set [75]. For the sake of brevity, it is denoted henceforth as aVQZ. The RIJK density fitting (DF) approximation [76] was used to accelerate the convergence of the SCF components of DLPNO-CCSD(T) energy. The corresponding auxiliary basis sets (aug-cc-pVQZ/JK and aug-cc-pVQZ/C in the ORCA nomenclature) [66] were used in the DF calculations of the SCF and correlation energies.

All computational approaches for the calculation of $\Delta_{f}H^{0}\left(g\right)$ employed in the present work were benchmarked on a series of energetic N-rich heterocycles and exhibited an average “chemical” accuracy (~4 kJ mol^−1^) [49,54,55].

Mechanical sensitivities were determined in accordance with the STANAG standards for the impact [77] and friction [78] sensitivity evaluation. Computational estimations of the detonation parameters were performed using the Pepekin–Lebedev empirical method [79]. This method is similar to the Kamlet approach, however, exhibits a better accuracy when compared with the available experimental data for the CHNO and CNO explosives (see more details in the Supporting Information of [21]).

The X-ray diffraction data were collected at 100 K on a Bruker Quest D8 diffractometer equipped with a Photon-III area-detector (graphite monochromator, ω-scan technique), using Mo K-radiation (0.71073 Å). The intensity data were integrated by the SAINT program [80] and were corrected for absorption and decay using SADABS [81]. The structure was solved by direct methods using SHELXS and was refined on F^2^ using SHELXL-2018 [82,83,84]. All atoms were refined with the particular anisotropic displacement parameters. The SHELXTL program suite was used for molecular graphics.

## 4. Conclusions

In the present work, we thoroughly characterized the thermal stability, mechanical sensitivity, and detonation properties for a wide set of 20 functionalized energetic furoxan derivatives. Differential scanning calorimetry revealed that the melting points of the species studied vary drastically in a broad range from ca. 40 °C (e.g., **2** and **14**) to 172 °C (**20**). This fact gives evidence that the phase-change properties of the furoxan derivatives can efficiently be tuned by introducing different substituents in the furoxan ring and/or the linkers between the two rings in the bicyclic species. Several monocyclic furoxans, viz., **1**, **4**, and **14,** showed no evidence of thermal decomposition below 180 °C and therefore are promising for applications. Although the estimated energetic performance (viz., detonation velocity and the heat of explosion) for these species is much higher than that of benchmark melt-cast explosives (TNT, DNAN), they are more volatile in comparison with the latter compounds. The study of the thermal behavior also showed that all 3-nitro furoxan derivatives (**2**, **4**–**7**, Scheme 2) exhibit an exothermic transformation near 120 °C. This fact was attributed to the thermal isomerization of the 3-nitrofuroxan moiety to the more thermodynamically preferable 4-nitrofuroxans.

The mechanical sensitivity of the furoxan derivatives varied from a low-to-insensitive level (**14**) to the highly sensitive bicyclic furoxans **3** and **18**–**20** (Table 2, Figure 6). From the safety viewpoint of complementary thermal stability and mechanical insensitivity, the most promising compounds are **4** and **14**. They can find applications in the melt-cast explosive formulations. At the same time, the compound **17** exhibits the same thermal and energetic parameters as a benchmark PETN explosive, whereas its sensitivity to impact and friction is slightly lower (Table 2, Figure 6). In turn, 3-methylfuroxan **15** is a remarkable mechanically insensitive energetic liquid that has a detonation velocity above 6 km s^−1^, at the cost of moderate thermal stability, though. Finally, the application prospects of DDF (**3**), one of the most powerful energetic materials synthesized up to date, are most probably limited due to the very high mechanical sensitivity of this compound.

## Figures and Tables

**Table 1:** Summary of the most reliable gas-phase formation enthalpies at 298 K (Δ*_f_H*^0^(*g*)) of furoxan and its derivatives (**1**–**27**, Figure 2, Figure 3 and Figure 9, Scheme 1, Scheme 2, Scheme 3 and Scheme 4), calculated using W2-F12 and W1-F12 multilevel procedures along with the atomization energy approach and domain-based local pair natural orbital (DLPNO)-CCSD(T)/aVQZ values of the enthalpies of isodesmic reactions. All values are given in kJ mol^−1^.

Compound	Δ*_f_H*^0^(*g*), kJ mol^−1^	Source
**1**	186.4	[a]
**2**	194.7	[a]
**3**	800.4	[b]
**4**	306.1	[b]
**5**	335.3	[c]
**6**	437.5	[d]
**7**	615.8	[c]
**8**	324.9	[c]
**9**	594.6	[c]
**10**	328.9	[c]
**11**	66.4	[a]
**12**	421.3	[a]
**13**	360.8	[a]
**14**	554.0	[a]
**15**	158.6	[a]
**16**	588.4	[b]
**17**	341.1	[b]
**18**	1053.2	[b]
**19**	479.9	[e]
**20**	552.6	[b]
**21**	205.2	[a]
**22**	246.6	[a]
**23**	237.1	[a]
**24**	418.2	[a]
**25**	425.6	[a]
**26**	697.3	[a]
**27**	699.5	[a]

[a] W2-F12 along with the atomization energy approach. [b] W1-F12 along with the atomization energy approach. [c] W1-F12 atomization energies of the reactants and DLPNO-CCSD(T) enthalpy of the isodesmic reactions (1), (3), and (4). The W1-F12 formation enthalpies Δ*_f_H*^0^(*g*) = 343.9 and 333.0 kJ mol^−1^ were used for **28** and **29**, respectively. [d] W2-F12 atomization energies of the reactants and DLPNO-CCSD(T) enthalpy of the isodesmic reaction (2). [e] W1-F12 atomization energies of the reactants and DLPNO-CCSD(T) enthalpy of the isodesmic reaction (5).

**Table 2:** The summary of the physical, safety, and energetic properties of the compounds **1**–**20** along with the reference energetic materials: trinitrotoluene (TNT), pentaerythritol tetranitrate (PETN), and hexogen (RDX). All data, except otherwise noted, are from the present work.

#	Stoichiometry	OB ^a^	ρ, g cm^−3^	Δ*_f_H*^0^(*s*), kJ mol^−1^	*t*_m_, °C	*t*_dec_, °C	IS, J	FS, N	*V*_D_, km s^−1^	*Q*_ex_, kJ g^−1^
**1**	C_3_H_3_N_3_O_4_	0.53	1.66	101 ^c^	65	188	5	270	7.8	5.4
**2**	C_3_H_3_N_3_O_4_	0.53	1.68	136	40	176	7	180	7.9	5.6
**3**	C_4_N_8_O_8_	1.00	1.98 ^b^	668 ^d^	–	117	0.7	6	10.0	7.4
**4**	C_5_H_5_N_3_O_4_	0.32	1.62	243	61	211	23	170	7.3	5.4
**5**	C_8_H_4_N_4_O_6_	0.33	1.60	267	90	162	2	>360	6.9	4.6
**6**	C_5_H_3_N_5_O_6_	0.52	1.76	365	103	129	3	70	8.2	5.8
**7**	C_10_H_3_N_7_O_10_	0.47	1.70	527	–	199	5	150	7.6	5.3
**8**	C_8_H_4_N_4_O_6_	0.33	1.58	258	83	175	8	>360	6.8	4.5
**9**	C_10_H_3_N_7_O_10_	0.47	1.77	518	133	173	3	150	7.8	5.3
**10**	C_8_H_3_N_5_O_8_	0.46	1.75	257	111	153	4	190	7.6	5.1
**11**	C_3_H_2_N_4_O_5_	0.71	1.85	−6	114	114	18	180	8.4	5.1
**12**	C_3_N_4_O_4_	0.67	1.87	381 ^d^	46	133	5	130	9.1	6.4
**13**	C_3_H_2_N_4_O_2_	0.29	1.90	288	117	96	45	90	8.5	4.9
**14**	C_4_N_4_O_2_	0.25	1.59	465 ^d^	39	204	>100	280	7.4	4.3
**15**	C_3_H_4_N_2_O_2_	0.25	1.24	70 ^e^	<RT	180	>100	–	6.1	4.1
**16**	C_6_H_6_N_6_O_4_	0.27	1.52	510	–	145	13	160	7.2	5.0
**17**	C_6_H_4_N_8_O_6_	0.43	1.88	254	–	188	4	120	8.3	4.5
**18**	C_6_N_8_O_4_	0.33	1.72	970	–	168	3	90	8.3	5.2
**19**	C_7_H_8_N_8_O_8_	0.44	1.68	405	–	125	3	110	7.9	5.4
**20**	C_6_H_6_N_6_O_5_	0.33	1.58	469	172	172	4	210	7.5	5.2
**NG**	C_3_H_5_N_3_O_9_	1.06	1.59	−370	13 ^f^	143 ^g^	<0.3	–	7.8	5.9
**TNT**	C_7_H_5_N_3_O_6_	0.36	1.64	−75	81	306	30	>360	6.8	4.2
**PETN**	C_5_H_8_N_4_O_12_	0.86	1.78	−539	141	181	3	70	8.4	5.7
**RDX**	C_3_H_6_N_6_O_6_	0.67	1.8	62	204	204	8	140	8.9	5.5

^a^ The oxygen balance with respect to CO_2_ and H_2_O: OB = [O]/(2*[C]+[H]/2). ^b^ Ref. [33]. ^c^ Ref. [24]. ^d^ Ref. [58]. ^e^ **15** is liquid at room temperature, therefore, we used the value of Δ*_vap_H*^0^ = 70 kJ mol^−1^ obtained by a group increment method [60]. ^f^ Ref. [61]. ^g^ Ref. [62].

## Supplementary Materials

The following are available online. Table S1: Crystal data and structure refinement for **12**, Table S2: Atomic coordinates and equivalent isotropic displacement parameters for **12**, Table S3: Bond lengths [Å] and angles [°] for **12**, Table S4: Anisotropic displacement parameters for **12**, Table S5: Torsion angles [°] for **12**. Figure S1: Thermal cycling of **1**: first heating up to melting, cooling with crystallization and reheating with a single endothermic event, Figure S2: The DSC curves of **1** and **2** at atmospheric pressure (solid curves) and under elevated nitrogen pressure of 2.0 MPa (dashed curves) at the heating rate at 5 K min^−1^. Figure S3: DSC traces for investigated furoxans 1–20 acquired at 5 K min-1 heating rate. The red curves correspond to the elevated pressure (2.0 MPa), the blue curves—to the atmospheric pressure. Table S6: A full set of the safety properties of the compounds studied.
