# Supplementary material for: Nitro-, Cyano-, and Methylfuroxans, and Their Bis-Derivatives: From Green Primary to Melt-Cast Explosives
Source: Molecules. 2020 Dec 10;25(24):5836. doi: 10.3390/molecules25245836 (PMC7764251; doi:10.3390/molecules25245836)
Supplement: Supplementary file 1 [file molecules-25-05836-s001.pdf]

Supporting information

Nitro-, Cyano- and Methylfuroxans, and Their Bis-Derivatives: From Green Primary to Melt-Cast Explosives

Alexander A. Larin [1], Dmitry M. Bystrov [1], Leonid L. Fershtat [1], Alexey A. Konnov [1], Nina N. Makhova [1], Konstantin A. Monogarov [2], Dmitry B. Meerov [2], Igor N. Melnikov [2], Alla N. Pivkina [2], Vitaly G. Kiselev [2,3,4], Nikita V. Muravyev [2,*]

[1] Zelinsky Institute of Organic Chemistry RAS, 47 Leninsky Ave., 119991 Moscow, Russia; fershtat@bk.ru
[2] Semenov Federal Research Center for Chemical Physics, Russian Academy of Sciences, 4 Kosygina Str., 119991 Moscow, Russia; alla_pivkina@mail.ru
[3] Novosibirsk State University, 1 Pirogova Str., 630090 Novosibirsk, Russia
[4] Institute of Chemical Kinetics and Combustion SB RAS, 3 Institutskaya Str., 630090 Novosibirsk, Russia; vitaly.kiselev@kinetics.nsc.ru
* Correspondence: n.v.muravyev@ya.ru; Tel.: +7-499-137-8203

Contents

1. The crystallographic data of **12**. 2

2. The thermal behavior of the compounds studied in the present work 7

3. The summary on the safety, and energetic properties of the species studied 18

*1. The crystallographic data of **12**.*

X-ray diffraction data were collected at 100K on a Bruker Quest D8 diffractometer equipped with a Photon-III area-detector (graphite monochromator, ω-scan technique), using Mo K_{α}-radiation (0.71073 Å). The intensity data were integrated by the SAINT program[1] and were corrected for absorption and decay using SADABS.[2] The structure was solved by direct methods using SHELXS,[3,4] and was refined on F^2 using SHELXL-2018.[4,5] All atoms were refined with individual anisotropic displacement parameters. The SHELXTL program suite[4] was used for molecular graphics. The CCDC 2042355 contains the supplementary crystallographic data for **12**. These data can be obtained free of charge via http://www.ccdc.cam.ac.uk/conts/retrieving.html (or from the CCDC, 12 Union Road, Cambridge, CB21EZ, UK; or deposit@ccdc.cam.ac.uk).

1. Bruker. APEX-III. *Bruker AXS Inc.*, Madison, Wisconsin, USA., 2016.
2. Krause, L.; Herbst-Irmer, R.; Sheldrick, G. M.; Stalke, D. Comparison of silver and molybdenum microfocus X-ray sources for single-crystal structure determination. *J. Appl. Cryst.* **2015**, 48, 3–10. http://doi.org/10.1107/S1600576714022985
3. Sheldrick, G. M. A short history of SHELX. *Acta Cryst.* **2008**, A64, 112-122. http://dx.doi.org/10.1107/S0108767307043930
4. Sheldrick, G. M. SHELXT - Integrated space-group and crystal-structure determination. *Acta Cryst.* **2015**, A71, 3-8. http://doi.org/10.1107/S2053273314026370
5. Sheldrick, G. M. Crystal structure refinement with SHELXL. *Acta Cryst.* **2015**, C71, 3-8. http://dx.doi.org/10.1107/S2053229614024218

Table S1. Crystal data and structure refinement for **12**.

Empirical formula	C3 N4 O4	
Formula weight	156.07	
Temperature	100(2) K	
Wavelength	0.71073 Å	
Crystal system	Orthorhombic	
Space group	$Pna2_1$	
Unit cell dimensions	a = 8.5661(3) Å	α= 90°.
	b = 6.1428(2) Å	β= 90°.
	c = 10.5647(3) Å	γ = 90°.
Volume	555.91(3) $Å^3$	
Z	4	
Density (calculated)	1.865 g/cm^3	
Absorption coefficient	0.175 mm^{-1}	
F(000)	312	
Crystal size	0.550 x 0.100 x 0.100 mm^3	
Theta range for data collection	3.837 to 29.994°.	
Index ranges	-12<=h<=12, -8<=k<=8, -14<=l<=14	
Reflections collected	12895	
Independent reflections	1614 [R(int) = 0.0330]	
Observed reflections	1564	
Completeness to theta = 25.242°	100.0 %	
Refinement method	Full-matrix least-squares on F^2	
Data / restraints / parameters	1614 / 1 / 100	
Goodness-of-fit on F^2	1.139	
Final R indices [I>2sigma(I)]	R1 = 0.0252, wR2 = 0.0616	
R indices (all data)	R1 = 0.0266, wR2 = 0.0630	
Absolute structure parameter	0.1(4)	
Largest diff. peak and hole	0.181 and -0.209 e.$Å^{-3}$	

Table S2. Atomic coordinates (x 10^4) and equivalent isotropic displacement parameters ($Å^2$x 10^3) for dbnitcn. U(eq) is defined as one third of the trace of the orthogonalized U^{ij} tensor.

	x	y	z	U(eq)
O(1)	6382(1)	8057(2)	6483(1)	16(1)
C(1)	7042(2)	5245(2)	5233(1)	12(1)
N(1)	5589(2)	8319(2)	5374(1)	16(1)
O(2)	8102(1)	5523(2)	7281(1)	18(1)
C(2)	5984(2)	6695(2)	4657(1)	13(1)
N(2)	7310(1)	6058(2)	6391(1)	13(1)
O(3)	4263(1)	7750(2)	3116(1)	19(1)
C(3)	7789(2)	3281(3)	4877(2)	16(1)
N(3)	8438(2)	1687(3)	4683(1)	22(1)
O(4)	5911(2)	5121(2)	2715(1)	19(1)
N(4)	5328(2)	6518(2)	3394(1)	14(1)

Table S3. Bond lengths [Å] and angles [°] for dbnitcn.

O(1)-N(1)	1.3644(18)
O(1)-N(2)	1.4659(16)
C(1)-N(2)	1.341(2)
C(1)-C(2)	1.409(2)
C(1)-C(3)	1.416(2)
N(1)-C(2)	1.2976(19)
O(2)-N(2)	1.2050(18)
C(2)-N(4)	1.4525(19)
O(3)-N(4)	1.2209(18)
C(3)-N(3)	1.144(2)
O(4)-N(4)	1.2251(18)
N(1)-O(1)-N(2)	108.19(10)
N(2)-C(1)-C(2)	105.56(13)
N(2)-C(1)-C(3)	118.84(14)
C(2)-C(1)-C(3)	135.59(14)
C(2)-N(1)-O(1)	106.26(12)
N(1)-C(2)-C(1)	113.73(14)

N(1)-C(2)-N(4)	119.49(13)
C(1)-C(2)-N(4)	126.77(13)
O(2)-N(2)-C(1)	134.94(14)
O(2)-N(2)-O(1)	118.80(12)
C(1)-N(2)-O(1)	106.25(11)
N(3)-C(3)-C(1)	174.65(17)
O(3)-N(4)-O(4)	126.74(14)
O(3)-N(4)-C(2)	117.64(13)
O(4)-N(4)-C(2)	115.62(13)

Table S4. Anisotropic displacement parameters ($Å^2$x 10^3) for dbnitcn. The anisotropic displacement factor exponent takes the form: $-2\pi^2[h^2 a^{*2}U^{11} + ... + 2 h k a^* b^* U^{12}]$

	U^{11}	U^{22}	U^{33}	U^{23}	U^{13}	U^{12}
O(1)	16(1)	16(1)	17(1)	-5(1)	-1(1)	4(1)
C(1)	11(1)	13(1)	13(1)	-2(1)	1(1)	0(1)
N(1)	15(1)	15(1)	18(1)	-2(1)	-1(1)	1(1)
O(2)	18(1)	21(1)	14(1)	0(1)	-2(1)	0(1)
C(2)	10(1)	12(1)	16(1)	0(1)	1(1)	-1(1)
N(2)	12(1)	13(1)	14(1)	-2(1)	2(1)	0(1)
O(3)	15(1)	19(1)	22(1)	5(1)	-2(1)	2(1)
C(3)	17(1)	16(1)	14(1)	-2(1)	-1(1)	1(1)
N(3)	26(1)	22(1)	20(1)	-6(1)	-6(1)	7(1)
O(4)	21(1)	21(1)	16(1)	-4(1)	1(1)	2(1)
N(4)	12(1)	14(1)	15(1)	2(1)	0(1)	-2(1)

Table S5. Torsion angles [°] for dbnitcn.

N(2)-O(1)-N(1)-C(2)	-0.80(15)
O(1)-N(1)-C(2)-C(1)	0.57(17)
O(1)-N(1)-C(2)-N(4)	-179.82(12)
N(2)-C(1)-C(2)-N(1)	-0.08(18)
C(3)-C(1)-C(2)-N(1)	178.96(17)
N(2)-C(1)-C(2)-N(4)	-179.66(13)
C(3)-C(1)-C(2)-N(4)	-0.6(3)
C(2)-C(1)-N(2)-O(2)	-179.65(16)
C(3)-C(1)-N(2)-O(2)	1.1(3)
C(2)-C(1)-N(2)-O(1)	-0.41(15)
C(3)-C(1)-N(2)-O(1)	-179.64(12)
N(1)-O(1)-N(2)-O(2)	-179.85(13)
N(1)-O(1)-N(2)-C(1)	0.77(14)
N(1)-C(2)-N(4)-O(3)	-9.7(2)
C(1)-C(2)-N(4)-O(3)	169.82(14)
N(1)-C(2)-N(4)-O(4)	170.47(14)
C(1)-C(2)-N(4)-O(4)	-10.0(2)

2. The thermal behavior of the compounds studied in the present work

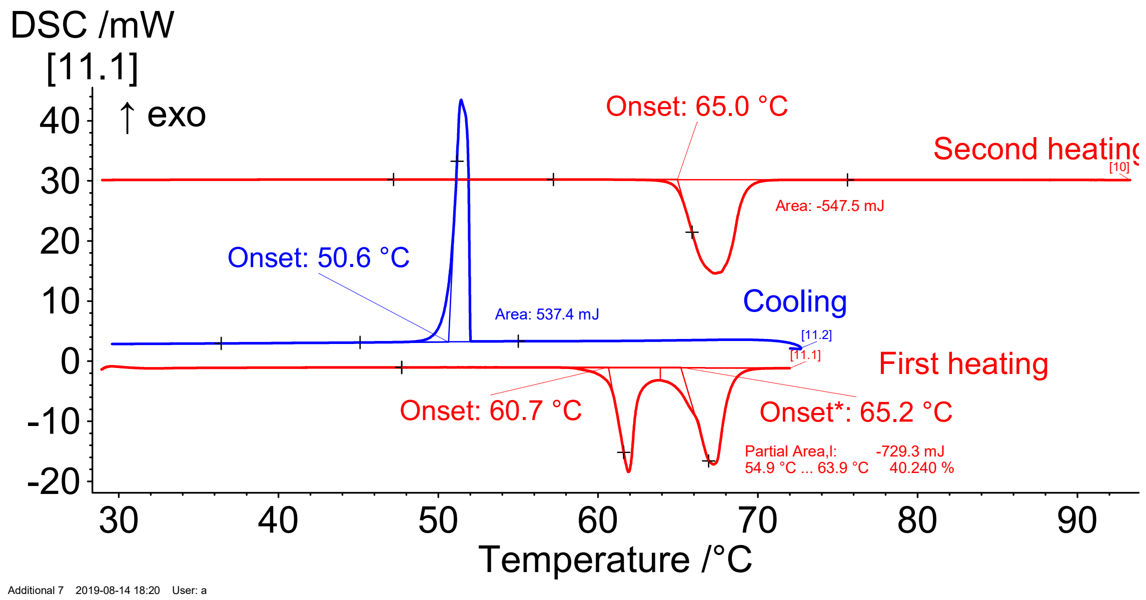

Figure S1. Thermal cycling of **1**: the first heating up to melting, cooling with crystallization, and reheating with a single endothermic event.

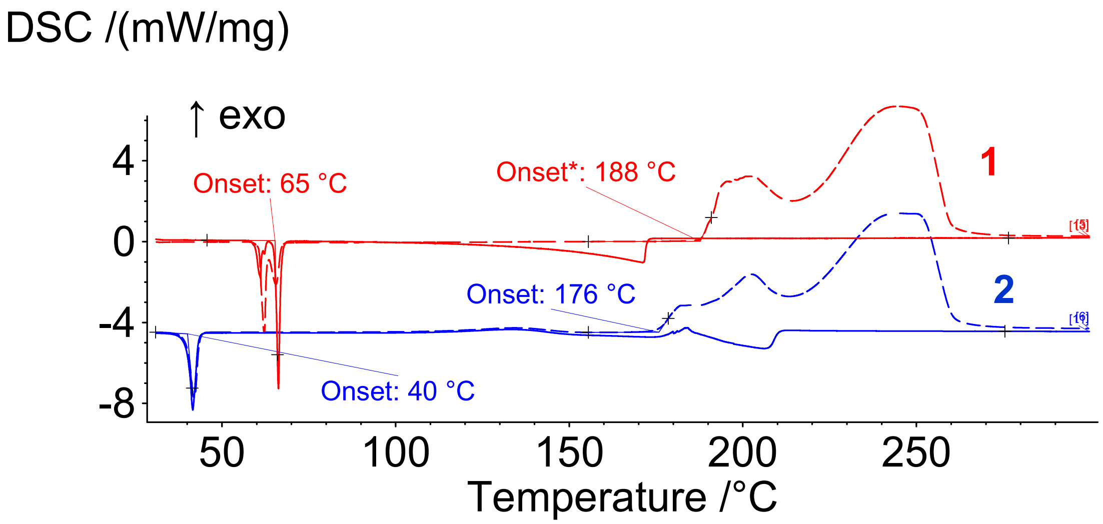

Figure S2. The DSC curves of **1** and **2** under atmospheric pressure (solid curves) and under elevated nitrogen pressure of 2.0 MPa (dashed curves) at the heating rate at 5 K min^{-1}.

Figure S3. DSC traces for all furoxan derivatives studied (**1** – **20**) acquired at 5 K min^{-1} heating rate. The red curves correspond to the elevated external pressure (2.0 MPa), the blue curves – to the atmospheric pressure.

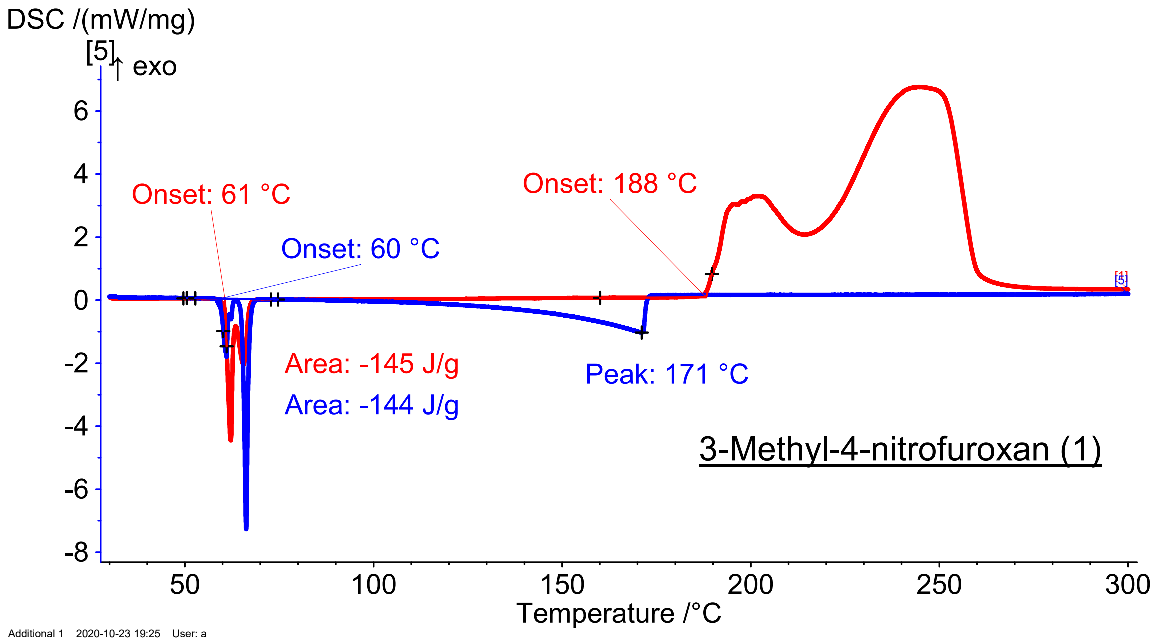

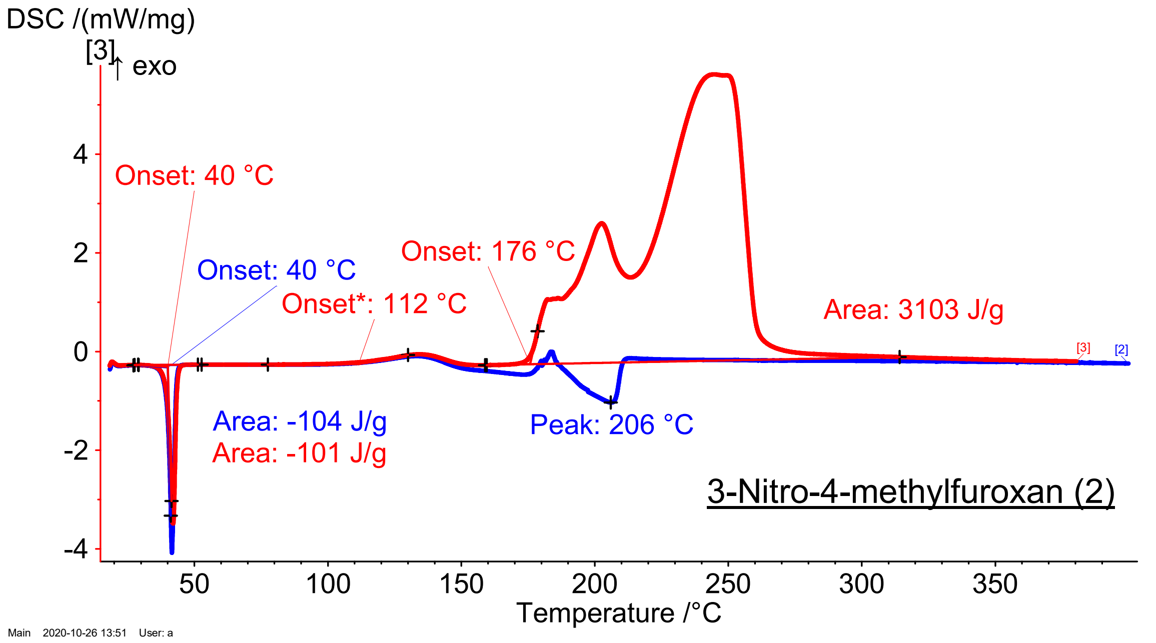

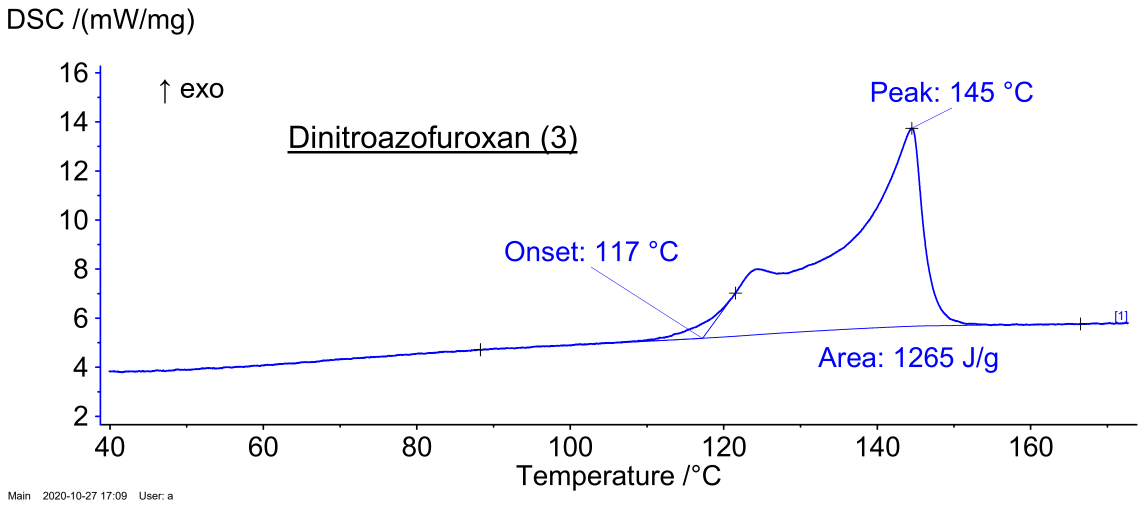

DSC /(mW/mg)
16
14
12
10
8
6
4
2
↑ exo
Dinitroazofuroxan (3)
Peak: 145 °C
Onset: 117 °C
[1]
Area: 1265 J/g
40
60
80
100
120
140
160
Temperature /°C
Main 2020-10-27 17:09 User: a

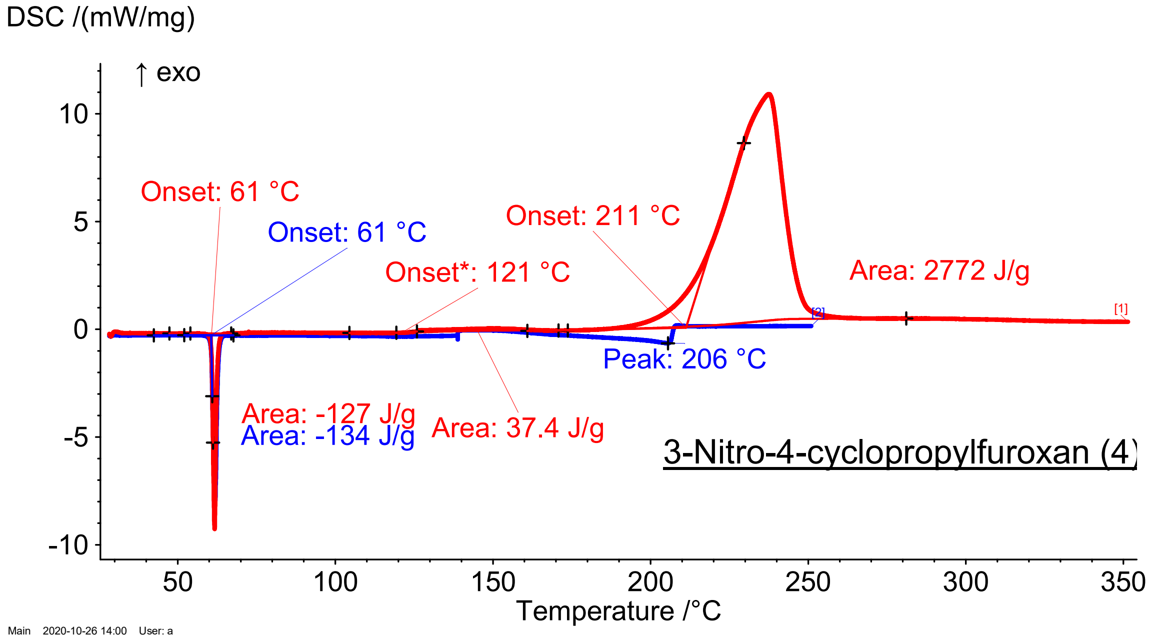

DSC /(mW/mg)
↑ exo
10
5
0
-5
-10
Onset: 61 °C
Onset: 61 °C
Onset: 211 °C
Onset*: 121 °C
Area: 2772 J/g
[1]
Peak: 206 °C
Area: -127 J/g
Area: -134 J/g
Area: 37.4 J/g
3-Nitro-4-cyclopropylfuroxan (4)
50
100
150
200
250
300
350
Temperature /°C
Main 2020-10-26 14:00 User: a

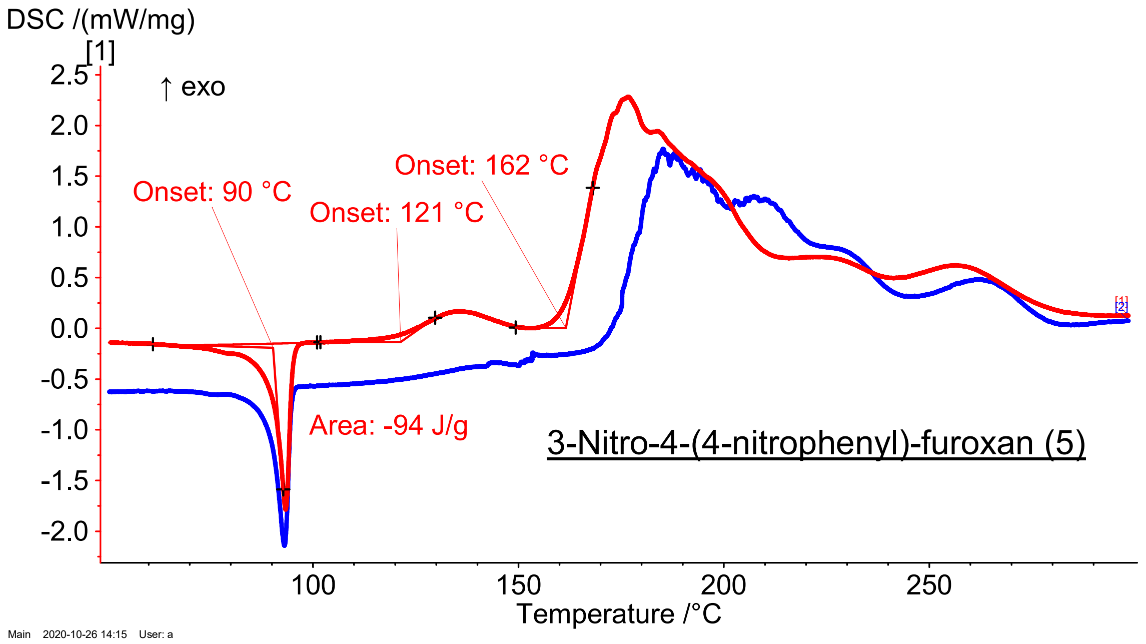
DSC /(mW/mg)
[1]
↑ exo
Onset: 90 °C
Onset: 121 °C
Onset: 162 °C
Area: -94 J/g
3-Nitro-4-(4-nitrophenyl)-furoxan (5)
Temperature /°C
Main 2020-10-26 14:15 User: a

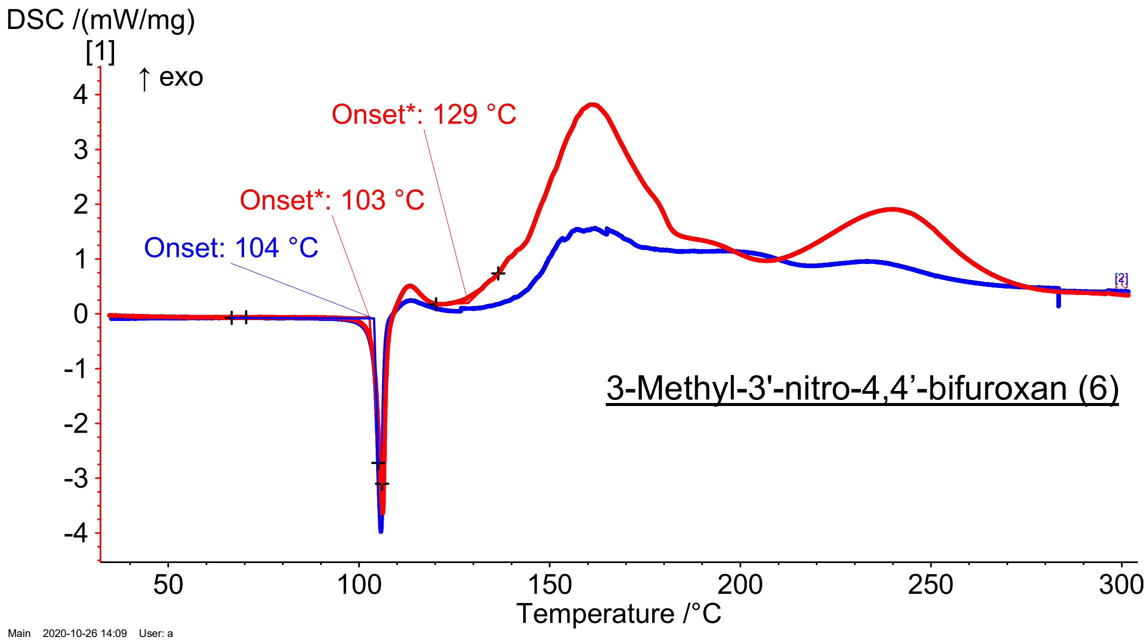
DSC /(mW/mg)
[1]
↑ exo
Onset*: 129 °C
Onset*: 103 °C
Onset: 104 °C
3-Methyl-3'-nitro-4,4’-bifuroxan (6)
Temperature /°C
Main 2020-10-26 14:09 User: a

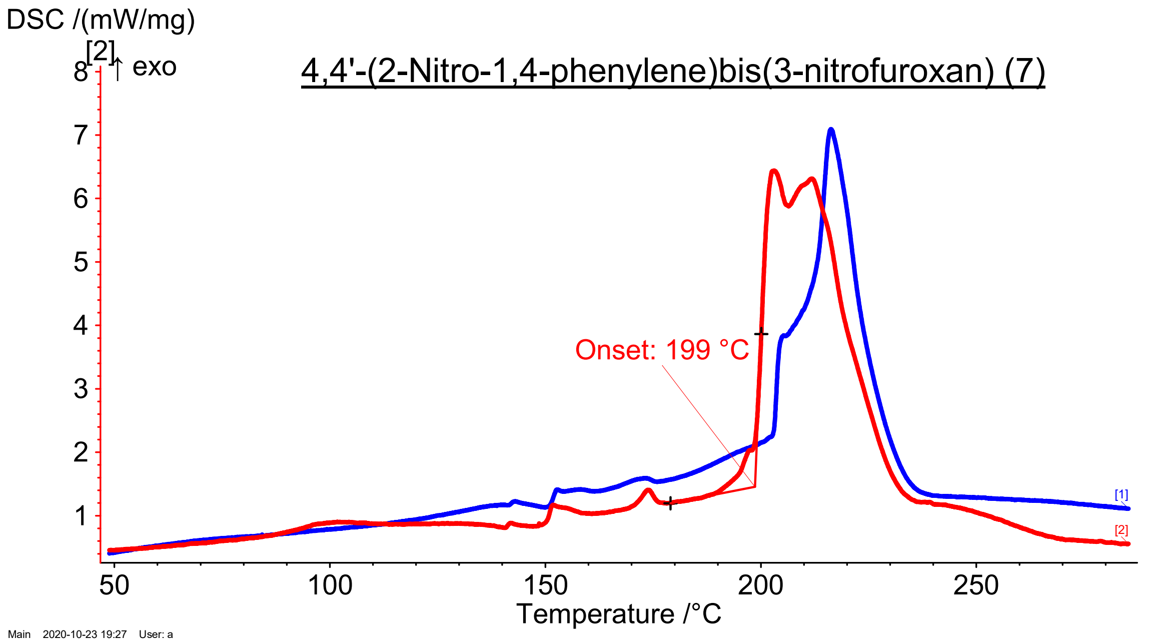
DSC /(mW/mg)
[2] ↑ exo
4,4'-(2-Nitro-1,4-phenylene)bis(3-nitrofuroxan) (7)
Onset: 199 °C
[1]
[2]
Temperature /°C
Main 2020-10-23 19:27 User: a

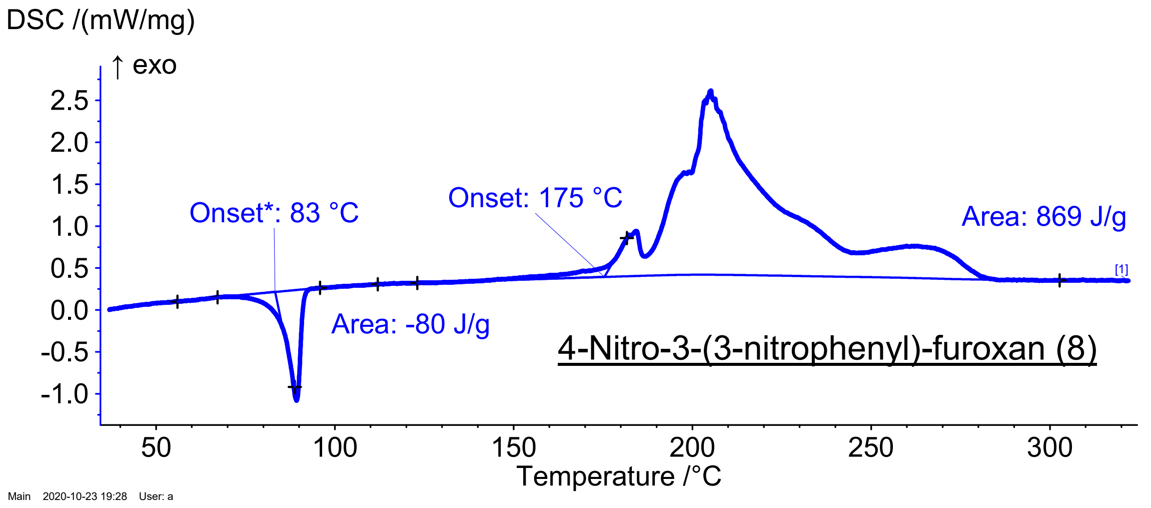
DSC /(mW/mg)
↑ exo
Onset*: 83 °C
Onset: 175 °C
Area: 869 J/g
[1]
Area: -80 J/g
4-Nitro-3-(3-nitrophenyl)-furoxan (8)
Temperature /°C
Main 2020-10-23 19:28 User: a

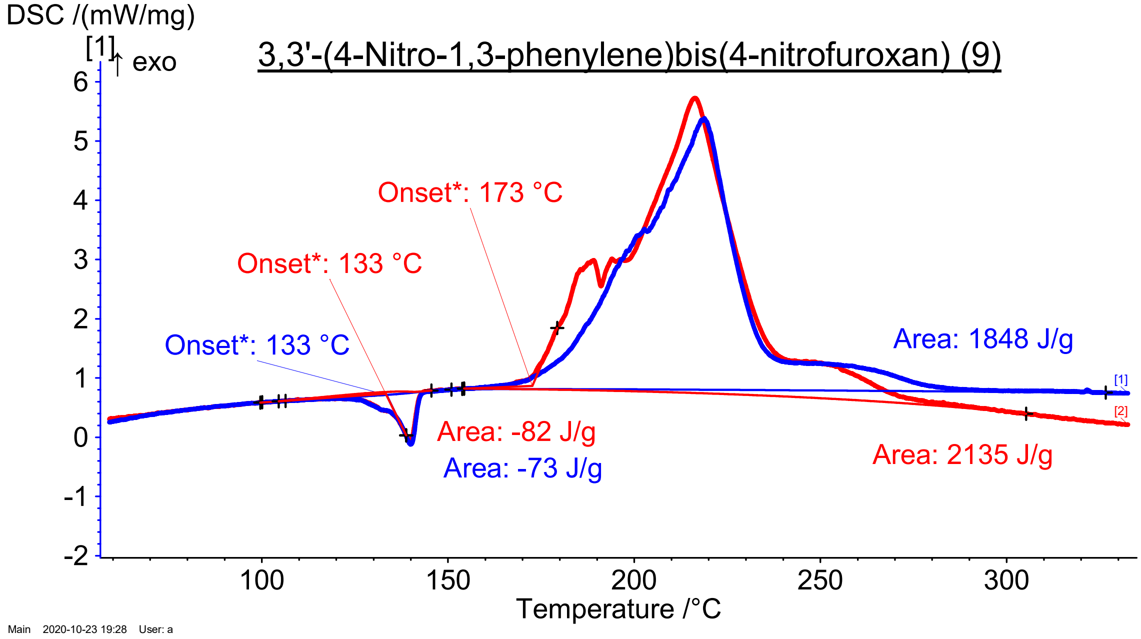
DSC /(mW/mg)
[1]
↑ exo
3,3'-(4-Nitro-1,3-phenylene)bis(4-nitrofuroxan) (9)
Onset*: 173 °C
Onset*: 133 °C
Onset*: 133 °C
Area: 1848 J/g
Area: -82 J/g
Area: -73 J/g
Area: 2135 J/g
[1]
[2]
Temperature /°C
Main 2020-10-23 19:28 User: a

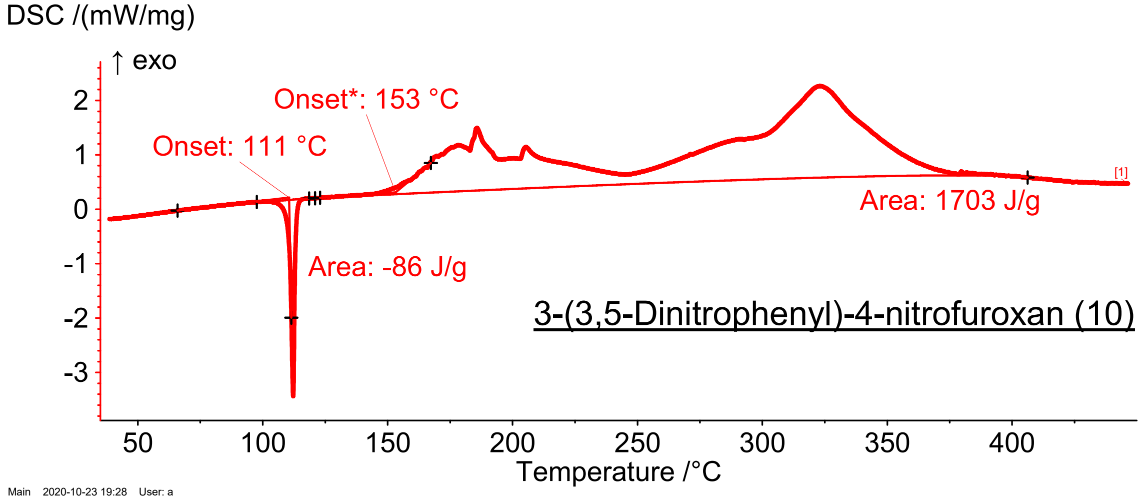
DSC /(mW/mg)
↑ exo
Onset*: 153 °C
Onset: 111 °C
Area: -86 J/g
Area: 1703 J/g
[1]
3-(3,5-Dinitrophenyl)-4-nitrofuroxan (10)
Temperature /°C
Main 2020-10-23 19:28 User: a

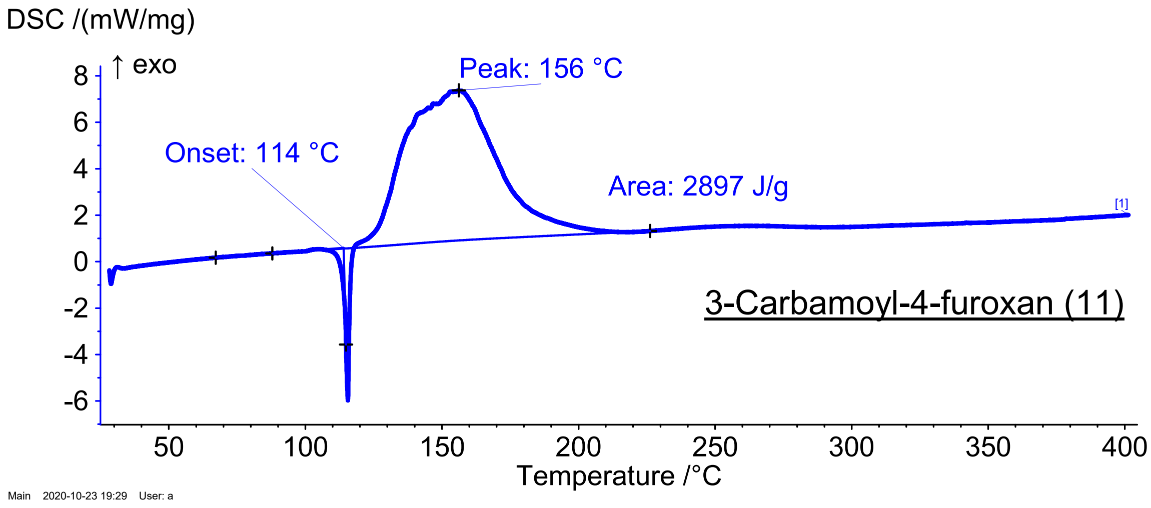
DSC /(mW/mg)
↑ exo
Peak: 156 °C
Onset: 114 °C
Area: 2897 J/g
[1]
3-Carbamoyl-4-furoxan (11)
8
6
4
2
0
-2
-4
-6
50
100
150
200
250
300
350
400
Temperature /°C
Main 2020-10-23 19:29 User: a

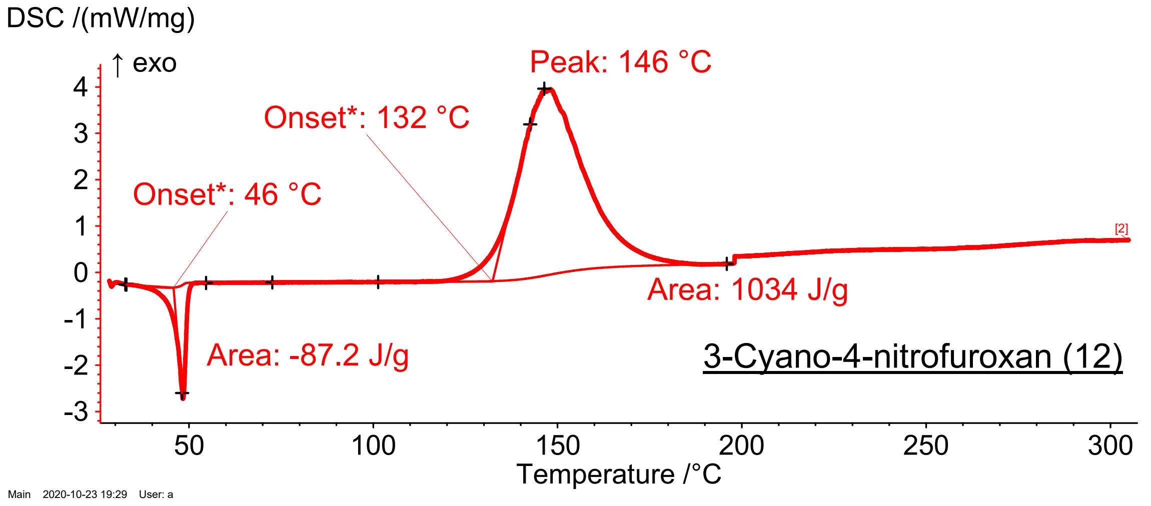
DSC /(mW/mg)
↑ exo
Peak: 146 °C
Onset*: 132 °C
Onset*: 46 °C
Area: 1034 J/g
[2]
Area: -87.2 J/g
3-Cyano-4-nitrofuroxan (12)
4
3
2
1
0
-1
-2
-3
50
100
150
200
250
300
Temperature /°C
Main 2020-10-23 19:29 User: a

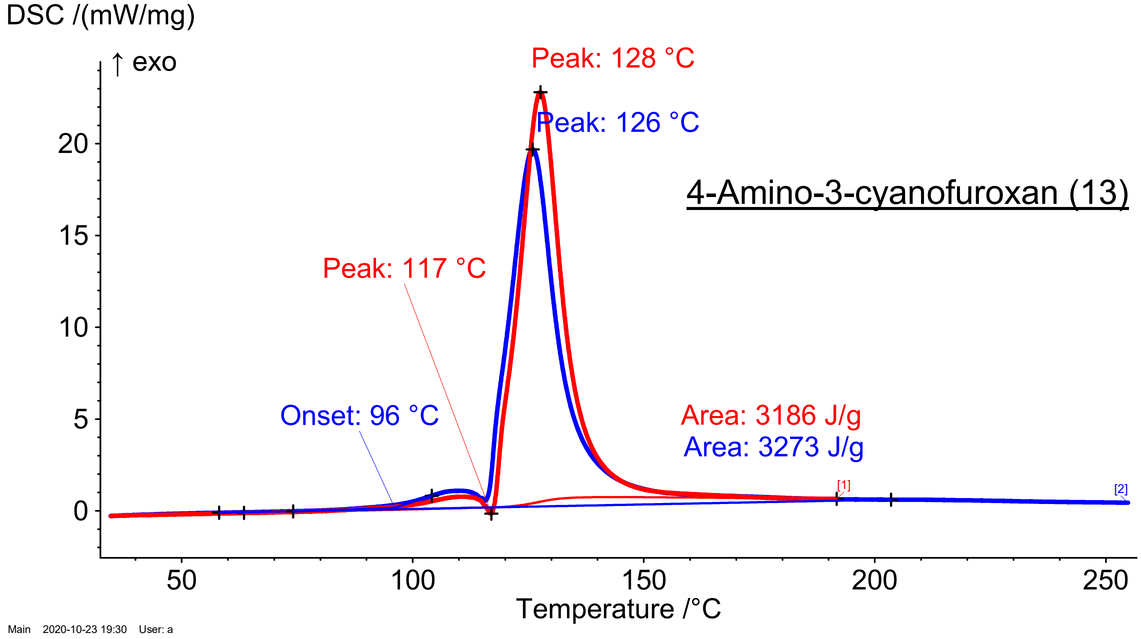
DSC /(mW/mg)
↑ exo
Peak: 128 °C
Peak: 126 °C
4-Amino-3-cyanofuroxan (13)
Peak: 117 °C
Onset: 96 °C
Area: 3186 J/g
Area: 3273 J/g
[1]
[2]
20
15
10
5
0
50
100
150
200
250
Temperature /°C
Main 2020-10-23 19:30 User: a

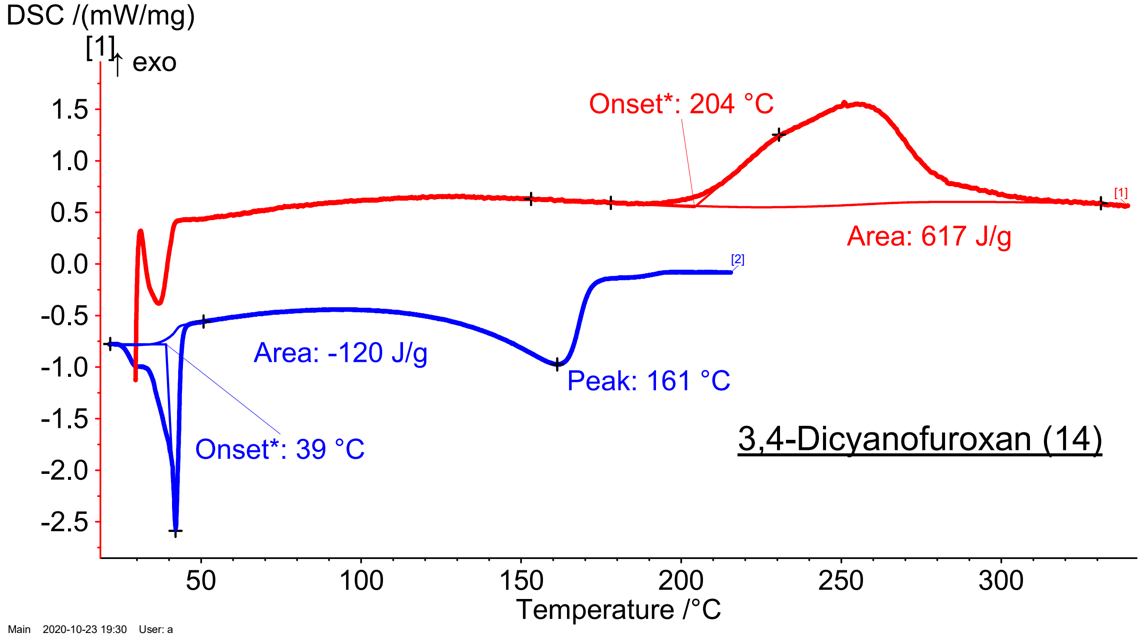
DSC /(mW/mg)
[1]
↑ exo
Onset*: 204 °C
[1]
Area: 617 J/g
[2]
Area: -120 J/g
Peak: 161 °C
Onset*: 39 °C
3,4-Dicyanofuroxan (14)
1.5
1.0
0.5
0.0
-0.5
-1.0
-1.5
-2.0
-2.5
50
100
150
200
250
300
Temperature /°C
Main 2020-10-23 19:30 User: a

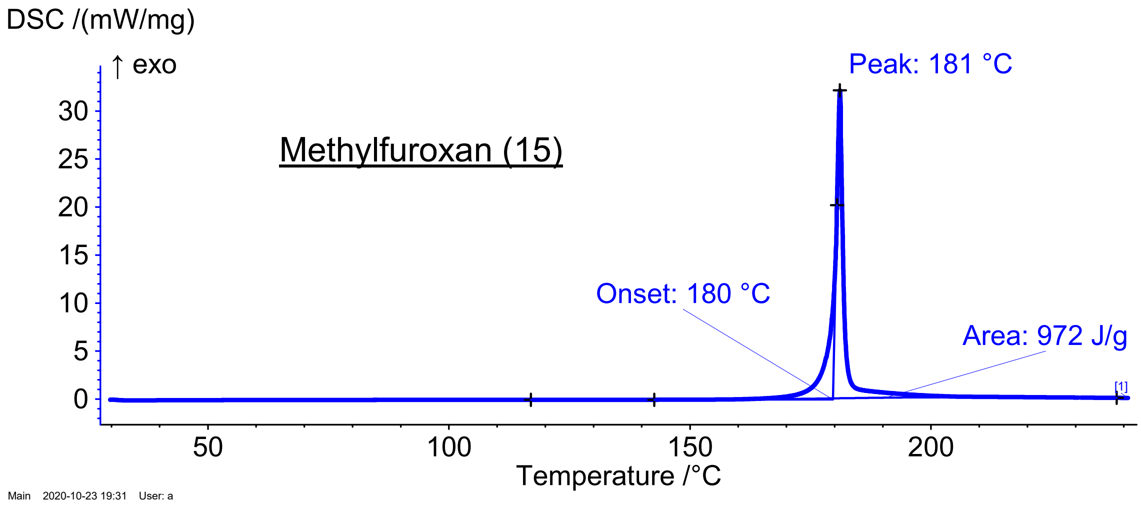

DSC /(mW/mg)
↑ exo
Methylfuroxan (15)
Peak: 181 °C
Onset: 180 °C
Area: 972 J/g
[1]
30
25
20
15
10
5
0
50
100
150
200
Temperature /°C
Main 2020-10-23 19:31 User: a

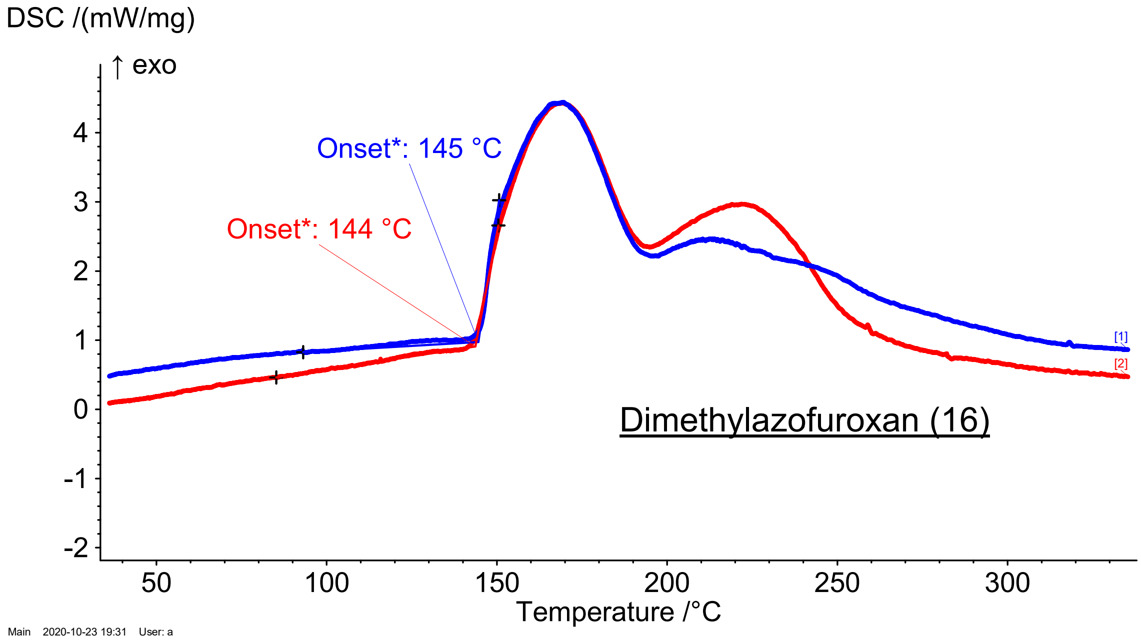

DSC /(mW/mg)
↑ exo
Onset*: 145 °C
Onset*: 144 °C
Dimethylazofuroxan (16)
[1]
[2]
4
3
2
1
0
-1
-2
50
100
150
200
250
300
Temperature /°C
Main 2020-10-23 19:31 User: a

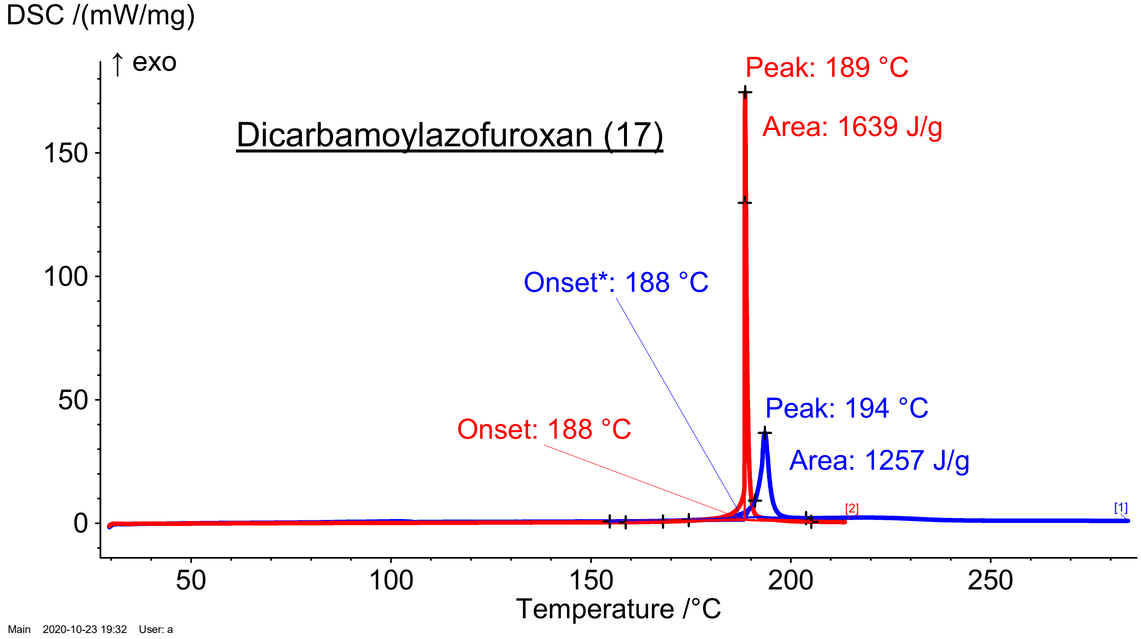
DSC /(mW/mg)
↑ exo
Dicarbamoylazofuroxan (17)
Peak: 189 °C
Area: 1639 J/g
Onset*: 188 °C
Peak: 194 °C
Onset: 188 °C
Area: 1257 J/g
Temperature /°C
Main 2020-10-23 19:32 User: a

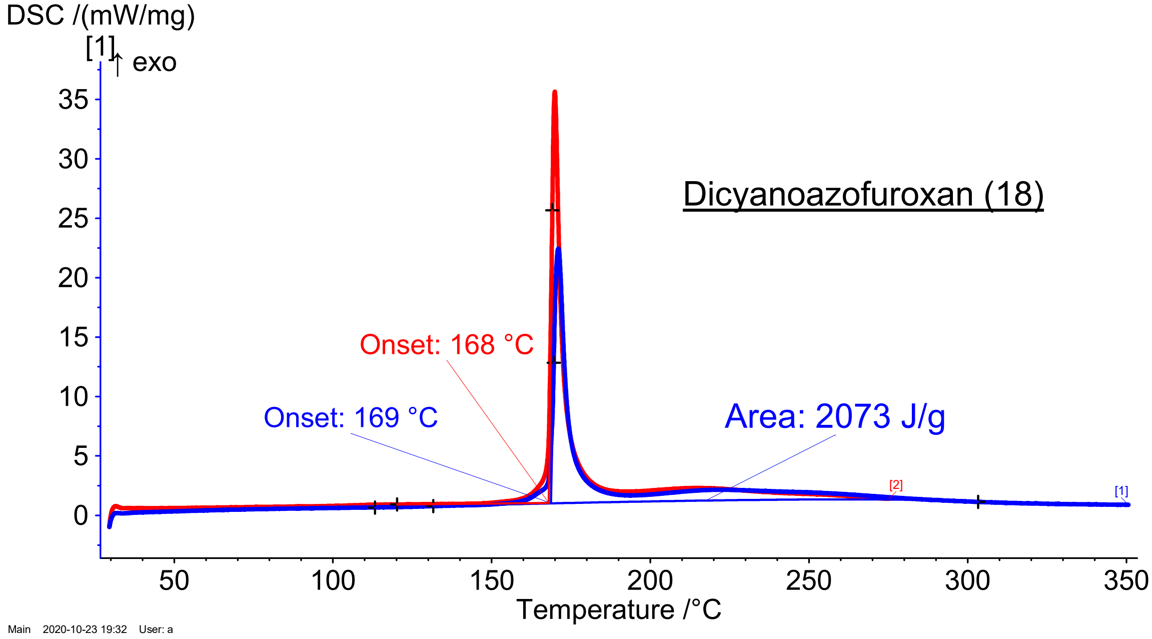
DSC /(mW/mg)
↑ exo
Dicyanoazofuroxan (18)
Onset: 168 °C
Onset: 169 °C
Area: 2073 J/g
Temperature /°C
Main 2020-10-23 19:32 User: a

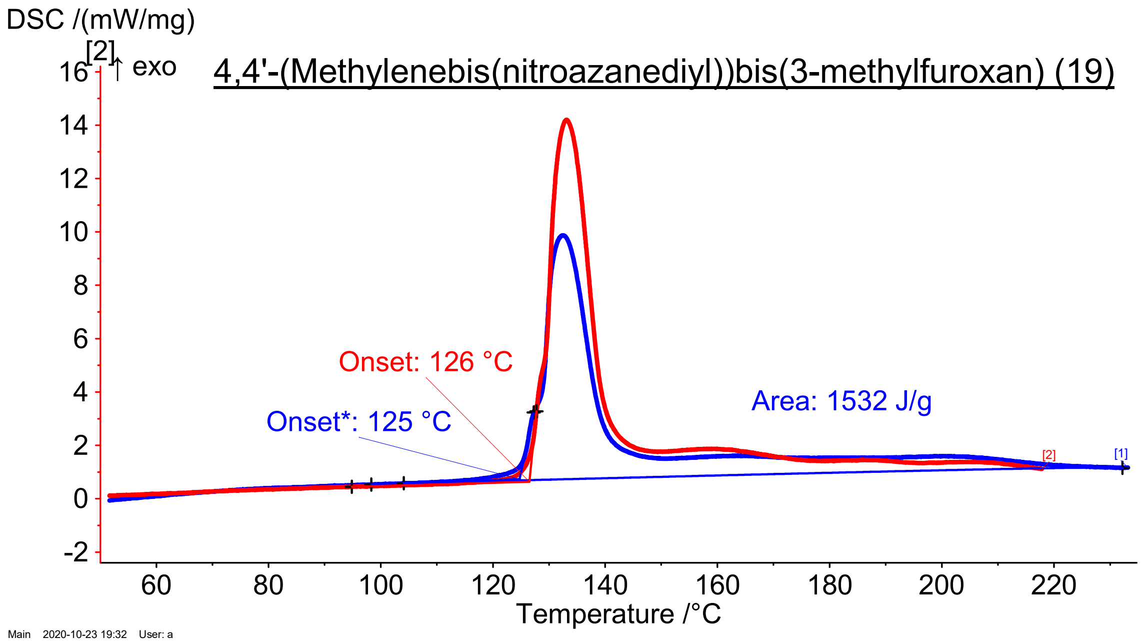
DSC /(mW/mg)
[2]
↑ exo
4,4'-(Methylenebis(nitroazanediyl))bis(3-methylfuroxan) (19)
Onset: 126 °C
Onset*: 125 °C
Area: 1532 J/g
[2]
[1]
Temperature /°C
Main 2020-10-23 19:32 User: a

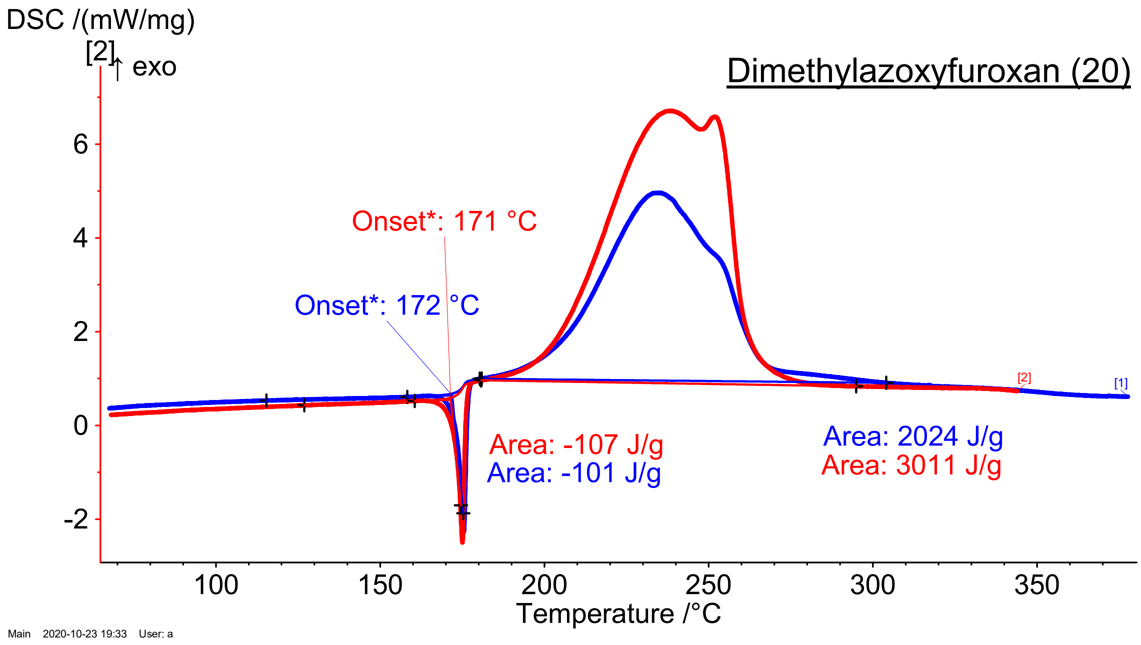
DSC /(mW/mg)
[2]
↑ exo
Dimethylazoxyfuroxan (20)
Onset*: 171 °C
Onset*: 172 °C
Area: -107 J/g
Area: -101 J/g
Area: 2024 J/g
Area: 3011 J/g
[2]
[1]
Temperature /°C
Main 2020-10-23 19:33 User: a

3. The summary of the safety and energetic properties of the species studied

Table S6. The working datasheets on the experimental properties of **1 – 20**.

#	Compound	Density, g cm-3[a]	Meltin g point, °C	Decomposition onset, °C [b]	Impact sensitivity, J	Friction sensitivity, N
**1**	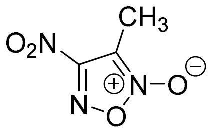 3-methyl-4-nitrofuroxan	1.66	60; 65	188 (p)	5.4 ± 2.1	273 ± 38
**2**	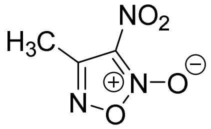 3-nitro-4-methylfuroxan	1.68	40	176 (p)	7.1 ± 1.0	179 ± 78
**3**	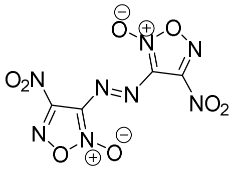 DDF, Dinitroazofuroxan	1.98 [Ref. 30]	-	117	0.7 ±0.2	5.9 ± 0.8
**4**	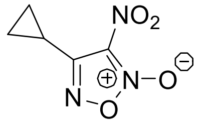 3-nitro-4-cyclopropylfuroxan	1.62	61	211 (p)	23 ± 11	173 ± 23
**5**	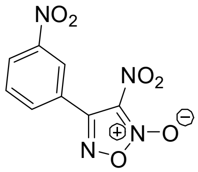 3-nitro-4-(4-nitrophenyl)-furoxan	1.60	90	162 (p)	2.1 ± 1.7	>360 (40%)

**6**	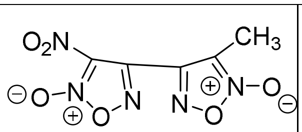 3-methyl-3'-nitro-4,4’-bifuroxan	1.76 (x)	103	129	2.5 ± 0.5	68 ± 31
**7**	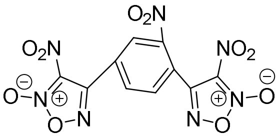 4,4'-(2-nitro-1,4-phenylene)bis(3-nitrofuroxan)	1.70	-	199	4.5 ± 0.3	149 ± 17
**8**	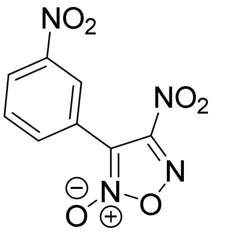 4-nitro-3-(3-nitrophenyl)-furoxan	1.58	83	175	7.6 ± 2.1	>360 (20 %)
**9**	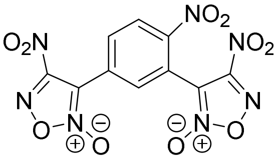 3,3'-(4-nitro-1,3-phenylene)bis(4-nitrofuroxan)	1.77	133	173 (p)	2.6 ± 0.5	150 ± 31
**10**	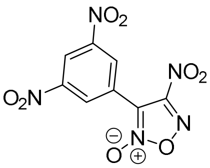 3-(3,5-dinitrophenyl)-4-nitrofuroxan	1.75	111	153	4.2 ± 0.3	194 ± 49
**11**	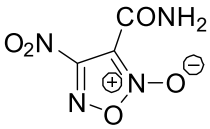 3-carbamoyl-4-	1.85	114	114	18 ± 5	176 ± 21

	nitrofuroxan					
**12**	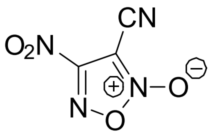 3-cyano-4-nitrofuroxan	1.87 (x)	46	132	4.5 ± 1.5	129 ± 19
**13**	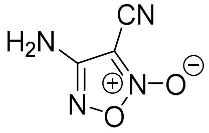 4-amino-3-cyanofuroxan	1.90	~117	96	45 ± 16	94 ± 10
**14**	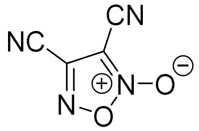 3,4-dicyanofuroxan	1.59	39	204 (p)	> 100	282 ± 50
**15**	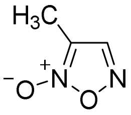 3-methylfuroxan	1.24	< RT	180	> 100	-
**16**	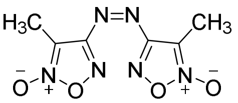 Dimethylazofuroxan	1.52	-	145	13 ± 3	158 ± 20
**17**	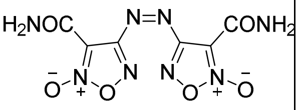 Dicarbamoylazofuroxan	1.88	-	188	3.5 ± 0.6	122 ± 18
**18**	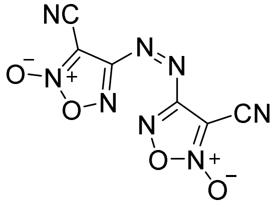	1.72	-	168	3.2 ± 0.3	86 ± 26

	Dicyanoazofuroxan					
**19**	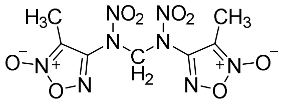<br>4,4'-(methylenebis(nitroazanediyl))bis(3-methylfuroxan)	1.68	-	125	3 ± 2	105 ± 22
**20**	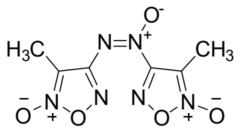<br>Dimethylazoxyfuroxan	1.58	172	172	4.4 ± 0.6	208 ± 37

Notes:

[a] The densities were determined using the gas picnometer except the values marked with (x) where the X-ray density is given.

[b] The extrapolated onset of the decomposition DSC peak was obtained at heating rate of 5 K min^{-1} and atmospheric pressure except the values denoted as (p), where the elevated pressure of 2.0 MPa was applied to suppress the vaporization of the sample.
